# Using intra-breath oscillometry in obesity hypoventilation syndrome to detect tidal expiratory flow limitation: a potential marker to optimize CPAP therapy

**DOI:** 10.1186/s12890-023-02777-x

**Published:** 2023-11-28

**Authors:** Szabolcs Baglyas, Luca Valkó, Vivien Móró, Eszter Podmaniczky, Dorottya Czövek, Gergely Makan, Zoltán Gingl, János Gál, Zoltán Hantos, András Lorx

**Affiliations:** 1 Department of Anesthesiology and Intensive Therapy, Semmelweis University, 1082 Üllői út 78/B, Budapest, Hungary; 2 Department of Pediatrics, Semmelweis University, Budapest, Hungary; 3 Department of Technical Informatics, University of Szeged, Szeged, Hungary

**Keywords:** Body position, Continuous positive airway pressure, Expiratory flow limitation, Obesity hypoventilation syndrome, Oscillometry

## Abstract

**Background:**

Continuous positive airway pressure (CPAP) therapy has profound effects in obesity hypoventilation syndrome (OHS). Current therapy initiation focuses on upper airway patency rather than the assessment of altered respiratory mechanics due to increased extrapulmonary mechanical load.

**Methods:**

We aimed to examine the viability of intra-breath oscillometry in optimizing CPAP therapy for OHS. We performed intra-breath oscillometry at 10 Hz in the sitting and supine positions, followed by measurements at increasing CPAP levels (none-5-10-15-20 cmH_2_O) in awake OHS patients. We plotted intra-breath resistance and reactance (*Xrs*) values against flow (*V’*) and volume (*V*) to identify tidal expiratory flow limitation (tEFL).

**Results:**

Thirty-five patients (65.7% male) completed the study. We found a characteristic looping of the *Xrs vs V’* plot in all patients in the supine position revealing tEFL: *Xrs* fell with decreasing flow at end-expiration. Intra-breath variables representing expiratory decrease of *Xrs* became more negative in the supine position [*end-expiratory Xrs* (mean ± SD): -1.9 ± 1.8 cmH_2_O·s·L^− 1^ sitting *vs*. -4.2 ± 2.2 cmH_2_O·s·L^− 1^ supine; *difference between end-expiratory and end-inspiratory Xrs*: -1.3 ± 1.7 cmH_2_O·s·L^− 1^ sitting vs. -3.6 ± 2.0 cmH_2_O·s·L^− 1^ supine, *p* < 0.001]. Increasing CPAP altered expiratory *Xrs* values and loop areas, suggesting diminished tEFL (*p* < 0.001). ‘Optimal CPAP’ value (able to cease tEFL) was 14.8 ± 4.1 cmH_2_O in our cohort, close to the long-term support average of 13.01(± 2.97) cmH_2_O but not correlated. We found no correlation between forced spirometry values, patient characteristics, apnea-hypopnea index and intra-breath oscillometry variables.

**Conclusions:**

tEFL, worsened by the supine position, can be diminished by stepwise CPAP application in most patients. Intra-breath oscillometry is a viable method to detect tEFL during CPAP initiation in OHS patients and tEFL is a possible target for optimizing therapy in OHS patients.

**Supplementary Information:**

The online version contains supplementary material available at 10.1186/s12890-023-02777-x.

## Background

Obesity hypoventilation syndrome (OHS), a leading cause of chronic respiratory failure worldwide, is characterized by obesity (body mass index [BMI] ≥ 30 kg·m^− 2^), and hypercapnia (arterial carbon dioxide ≥ 45 mmHg) that typically worsens at night [1, 2]. The pathophysiology of OHS is complex. Upper airway patency issues, disrupted control of breathing, and altered lung mechanics due to obesity may all contribute to the characteristic hypoventilation [3–5].

Due to this complex pathophysiology, optimal therapy for patients has not yet been clearly established. Current clinical guidance advises continuous positive airway pressure (CPAP) therapy intended to achieve daytime normocapnia, and in cases of insufficient clinical improvement, progression to bilevel ventilation [1]. CPAP counteracts the increased mechanical load on the respiratory system and the subsequent decrease in end-expiratory lung volume (EELV). Additionally, it manages upper airway patency issues, which are common in OHS patients [6, 7]. Bilevel ventilation may be useful in patients with exhausted respiratory drive or where upper airway obstruction is not prominent [8, 9]. Despite this practice, it is unclear how the “ideal CPAP” level can be determined and whether CPAP failure can be managed with a more optimized CPAP setting. There are currently no official titration protocols in use. Recent studies have used protocols where gradual increase of CPAP is continued even after obstructive events and flow limitation have ceased, if saturation goals are not reached [10, 11]. A recent study has shown the majority of patients with lower AHI (< 30/h) can still benefit from CPAP values of 8–14 cmH_2_O [11]. This effect is probably attributable to restored absolute lung volumes. While hypoventilation and upper airway patency issues can be assessed using polysomnography, changes in the absolute lung volume and subsequent airway dynamics are currently not evaluated during routine therapy induction for OHS. Optimizing therapy by restoring EELV might improve long-term outcomes in this patient population.

Small airway closure due to the reduction in absolute lung volumes during normal tidal breathing has been demonstrated using several methods in obese patients, including the negative expiratory pressure (NEP) technique and oscillometry [12–14]. Intra-breath oscillometry detects dynamic changes in large and small airways, as well as in peripheral inhomogeneity during tidal breathing [15]. As an additional benefit, tidal expiratory flow limitation (tEFL) can be revealed even during therapeutic intervention [16, 17].

The current study aimed to assess the viability of intra-breath oscillometry in optimizing CPAP therapy for OHS by measuring tEFL in awake OHS patients in different body positions and at different CPAP settings. We hypothesized that, using this method, we could accurately detect tEFL and its reversal when applying the “optimal CPAP” level.

## Methods

### Design of the study

We screened patients receiving therapy for OHS through the Semmelweis University Home Ventilation Program (Budapest, Hungary) from 05/01/2021 to 31/01/2022. After obtaining written informed consent, we collected demographic data and performed arterial blood gas analysis and forced spirometry tests. We then used intra-breath oscillometry in sitting and supine positions to assess the baseline tEFL of the study patients. We then performed a measurement in the supine position with stepwise application of CPAP to determine the CPAP level that could obliterate tEFL (e.g. “optimal CPAP”). We examined the correlation of tEFL with classic disease severity markers, such as forced spirometry test values, BMI, and apnea-hypopnea index (AHI). The local committee of Semmelweis University approved this study in agreement with the Scientific and Human Research Ethics Committee of the Hungarian Medical Research Council (SE TUKEB 239/2018).

### Inclusion and exclusion criteria

Adult (> 18 years) patients with previously confirmed OHS (as per the Task Force Report of the European Respiratory Society) [2] already established on intermittent CPAP or bilevel ventilation were eligible for the study. Exclusion criteria included unstable condition (worsening of respiratory symptoms in the previous 30 days), coexisting chronic obstructive lung disease, and chronic cough or sputum production.

### Data collection

The data collected at screening included diagnostic criteria, OHS stage, obstructive sleep apnoea (OSA) level, and long term ventilation therapy settings. The OHS stage was identified at the time of the initiation of long-term respiratory support. The OSA level was based on the AHI values recorded during the initial polysomnography at the time of diagnosis. We collected demographic data (including age, sex, and BMI) at the time of oscillometry and spirometry measurements. Arterial blood gas sampling was performed on room air at least 15 min after discontinuing contingent oxygen supplementation. Patients underwent forced spirometry tests following oscillometry in both body positions using the Piston PinkFlow spirometer (Piston Medical Ltd, Budapest, Hungary), according to the American Thoracic Society and European Respiratory Society guidelines [18]. The forced vital capacity (*FVC*), *FVC* predicted %, forced expiratory volume in 1 s (*FEV*_*1*_), *FEV*_*1*_ predicted %, and *FEV*_1_*/FVC* ratio were recorded.

### Oscillometry

Respiratory oscillometry employs external small-amplitude oscillations on spontaneous breathing while measuring the mechanical response of the respiratory system, expressed as respiratory impedance (*Zrs*). The two components of *Zrs* reflect the sum of the total airway and tissue resistances (resistance - *Rrs*) and the elastic and inertial components, describing the ability to store energy and promote passive exhalation (reactance – *Xrs*). In contrast to conventional multiple-frequency oscillometry determining mean Zrs over multiple breaths, the novel mono-frequency intra-breath modality follows the changes in *Rrs* and *Xrs with* volume *(V)* and flow *(V’)* within the breathing cycle [15]. The decrease in *Xrs* during expiration is a sensitive marker of the dynamic changes in small airway mechanical properties, reflecting tEFL [16, 17]. The difference between the mean values of expiratory and inspiratory reactance (*ΔXmean*) has been used by several studies to identify tEFL, however, measurements under CPAP therapy may require a more complex assessment of the *Xrs vs. V* and *Xrs vs. V’* relationships [16, 17, 19, 20]. In particular, the high CPAP levels used for OHS may result in glottal interference of impedance values, which might interfere with the ability of *ΔXmean* to accurately reflect tEFL [21]. To better characterize the *Xrs vs. V* and *Xrs vs. V’* relationships and possibly identify glottal interference at high CPAP levels, we measured a variety of intra-breath parameters (*see* Table 1). In addition to measuring *Zrs* variables at certain time points of the breathing cycle (*ReE, ReI, ΔR, XeE, XeI, ΔX*), we calculated established (*ΔXmean*) and newly proposed tEFL markers (*AXV* and, *AXV’*) [16].

**Table 1:** Definition of intra-breath oscillometry variables

Parameter	Unit	Definition
**Vt**	**Liter**	Tidal volume
**Frequency**	**min** ^**− 1**^	Breathing frequency
**Ti/Ttot**		Inspiratory time divided by breathing cycle time
**ReE**	**cmH**_**2**_ **O·s·L** ^**− 1**^	Respiratory resistance at the end of expiration
**ReI**	**cmH**_**2**_ **O·s·L** ^**− 1**^	Respiratory resistance at the end of inspiration
**ΔR**	**cmH**_**2**_ **O·s·L** ^**− 1**^	Difference between ReE and ReI
**ΔXmean**	**cmH**_**2**_ **O·s·L** ^**− 1**^	Difference between the mean values of expiratory and inspiratory reactance
**XeE**	**cmH**_**2**_ **O·s·L** ^**− 1**^	Respiratory reactance at the end of expiration
**XeI**	**cmH**_**2**_ **O·s·L** ^**− 1**^	Respiratory reactance at the end of inspiration
**ΔX**	**cmH**_**2**_ **O·s·L** ^**− 1**^	Difference between XeE and XeI
**AXV**	**cmH**_**2**_ **O·s**	Area of the reactance vs. volume diagram
**AXV’**	**cmH**_**2**_ **O**	Area of the reactance vs. flow diagram
**ARV**	**cmH**_**2**_ **O·s**	Area of the resistance vs. volume diagram
**ARV’**	**cmH**_**2**_ **O·s·L** ^**− 1**^	Area of the resistance vs. flow diagram

We measured *Zrs* using a custom-made oscillometry setup (Figure S1). The system consisted of a loudspeaker-in-box system for the generation of an oscillatory signal (frequency: 10 Hz, amplitude: 1 cmH_2_O), pressure sensors (Honeywell model 26PCAFA6D, Golden Valley, MN, USA), and a screen pneumotachograph to measure P and V’. We used a noninvasive respiratory device (A40; Philips Respironics, Murrysville, PA, USA) to generate CPAP.

We conducted oscillometry in awake patients during tidal breathing in sitting and supine positions. Before recording, we instructed the patients according to technical standards and applied a nose clip and cheek support [22]. The baseline recording was started when a consistent, homogeneous breathing pattern was established and lasted for 30 s. We then recorded for 120–240 s in the supine position with stepwise elevations of CPAP levels from 0 to 5, 10, 15, and 20 cmH_2_O. At each CPAP level, we selected 3–8 regular, artefact-free breathing cycles for intra-breath analysis. We discontinued measurements after two unsuccessful attempts in case of discomfort or intolerance (e.g., inability to maintain CPAP without leakage around the mouthpiece).

An optimal CPAP level was defined after the measurement during data processing. The discrete CPAP level was considered optimal when the typical anticlockwise looping of AXV’ diminished below the threshold as |*AXV’*| < 1 cmH_2_O.

**Fig. 1:**
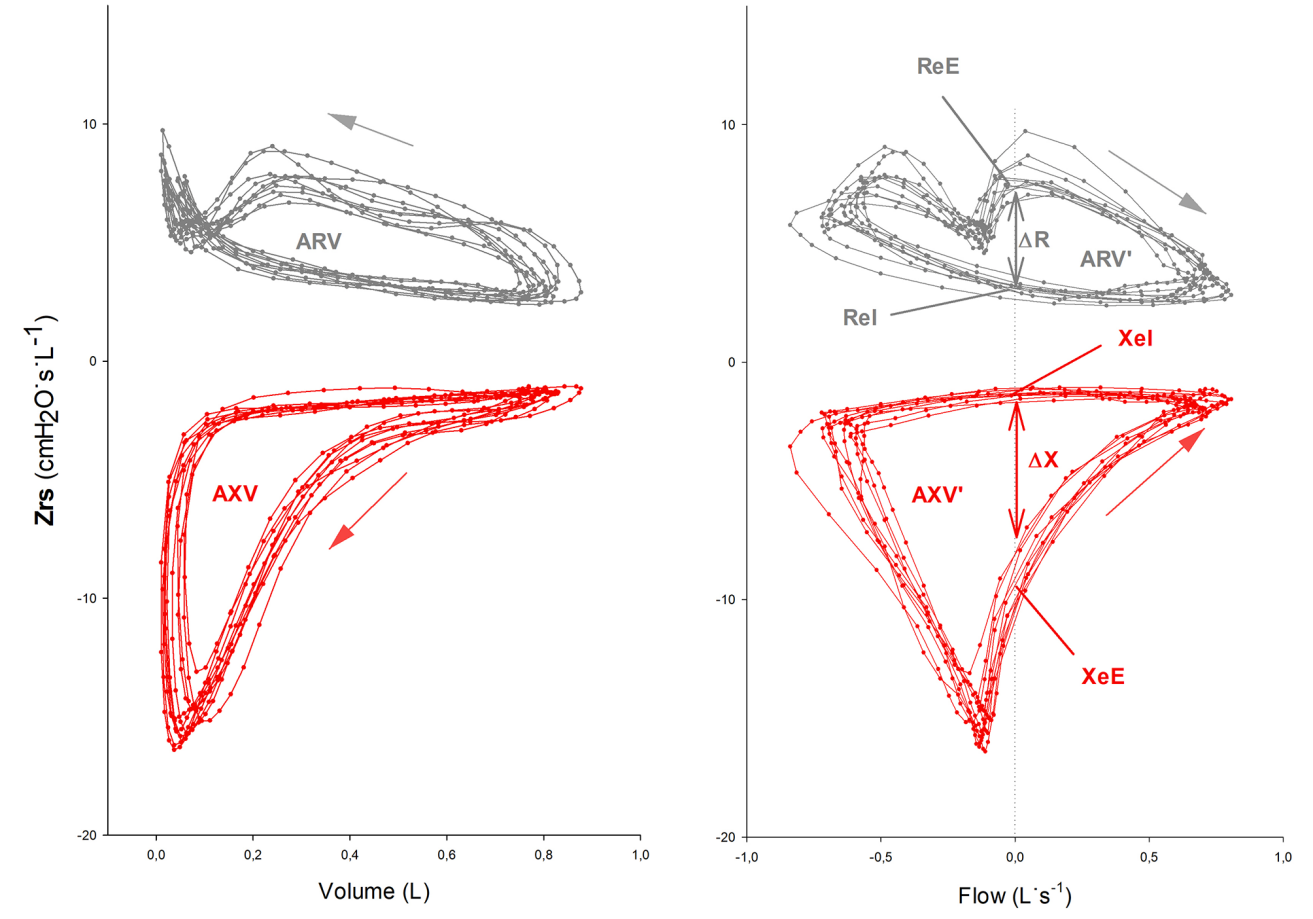
Measured and derived intra-breath oscillometry variables. The two parts of the respiratory impedance (*Zrs*), the resistance (*Rrs*) and the reactance (*Xrs*), are plotted in grey and red, respectively. *Rrs* reflects the sum of total airway and tissue resistances, whereas *Xrs*, which consists of elastic and inertial components, describes the ability to store energy and promote passive exhalation. A single patient’s *Rrs* and *Xrs* recordings are mapped against tidal volume and flow parameters. The arrows mark the direction of looping during a single breathing cycle. Positive flow indicates inspiration and negative flow indicates expiration. Plotting *Rrs* and *Xrs* against volume and flow permits insight into the intra-breath changes of airway mechanics in a detailed manner. To describe complex intra-breath changes in respiratory mechanics the following measured and derived variables were used: ReE = end expiratory resistance; ReI = end inspiratory resistance; ΔR = difference between ReE and ReI; XeE = end expiratory reactance; XeI = end inspiratory reactance; ΔX = difference between XeE and XeI; AXV = area of Xrs vs V diagram; AXV’ = area of Xrs vs. V’ diagram;

We computed the auto- and cross-correlation spectra of the recorded P and V’ signals using the fast Fourier transform algorithm and retained a bandwidth of ± 2 Hz at 10 Hz oscillation frequency for further analysis. V was obtained by numerical integration of V’. Definitions of measured and derived *intra-breath variables* are presented in Fig. 1; Table 1. We computed the values of *Rrs* and *Xrs* for each oscillation period (0.1 s) smoothed by a moving average over 0.5 s. We identified the different phases of the breathing cycle according to the interpolated zero crossings of V’. We determined the corresponding end-expiratory and end-inspiratory (i.e., zero-flow) *Rrs* values (*ReE* and *ReI*) and *Xrs* values (*XeE* and *XeI*), as well as their corresponding differences (*ΔR* and *ΔX*).

Intra-breath oscillometry variables were pooled and compared in the sitting and supine positions to identify tEFL worsening in the supine body position (characteristic of severe obesity). We then identified diminishing tEFL with stepwise application of CPAP. We calculated the correlation between previously used marker of tEFL (*ΔXmean from our 10-Hz data*) and the newly proposed parameters (*XeE*, *ΔX*, *AXV*, and *AXV’*).

We further analysed intra-breath oscillometry variables according to patient subgroups (OSA vs. no OSA and severe OSA vs. no severe OSA).

### Statistical analysis

Continuous variables are presented as the mean and standard deviation (SD), and categorical variables are presented as n (%). Intra-breath oscillometry variables (*ReE, ReI, ΔR, XeE, XeI, ΔX, ΔXmean, AXV, AXV’*) in the two positions were analysed with Wilcoxon signed rank test. We used the Mann-Whitney U test to compare demographic data (age, BMI, baseline AHI) and oscillometry parameters (*XeE, ΔX, AXV, AXV’*) between men and women. The effect of CPAP level on the measured intra-breath oscillometry variables was analysed using Friedman ANOVA and Kendall’s concordance tests. The correlation between oscillometry and spirometry parameters (*FEV*_*1*_, *FEV*_*1*_*%, FEV*_*1*_*/FVC*, and *XeE, ΔX, AXV, AXV’*) was analysed using the Spearman rank order correlation. We assessed the correlation between the ‘optimal’ CPAP and the CPAP or expiratory positive airway pressure (EPAP) used by patients during long term respiratory support. We identified subgroups within the study group based on the presence or absence of OSA (AHI ≥ 5/h or < 5/h) and the presence or absence of severe OSA (AHI ≥ 30/h or < 30/h). We used the Mann-Whitney U test to compare spirometry and oscillometry parameters (*XeE, ΔX, AXV, AXV’*) within the subgroups.

Statistical significance was set at *p* < 0.05. Data analysis was conducted using Statistica 13 software (Tibco Data Science, Hamburg, Germany). We did not use the missing data for the calculations. Figures were created using SigmaPlot 14.5 (Systat Software, San Jose, United States).

## Results

### Patients

Thirty-five patients were measured in the sitting and supine position, and 33 completed the CPAP measurements. The demographic data are summarized in Table 2. Mean BMI was 49.34 kg/m^2^ (range 32.33–91.05 kg/m^2^). Note that 88.6% of patients had severe (III-IV stage) OHS and that blood gas values reflected adequate treatment [23]. Demographic data (age, BMI, baseline AHI) did not differ in the two sexes (data not shown).

**Table 2:** Demographic data of study patients

		Mean (SD, or %)
**Male** N		23 (65.71%)
**Age** years		56.31 (± 8.63)
**BMI** kg/m^2^		49.34 (± 10.88)
**Baseline AHI** /h^†^		23.93 (± 28.08)
**AHI ≥ 5/h** N		24 (77.4%)
**AHI ≥ 30/h** N		7 (22.6%)
***OHS stage (according to Shah NM et al.)*** [23]		
	**I**	3 (8.6%)
	**II**	1 (2.9%)
	**III**	19 (54.3%)
	**IV**	12 (34.3%)
***Arterial blood gas*** ^***#***^		
	**pH**	7.42 (± 0.04)
	**pCO**_**2**_ mmHg	42.89 (± 8.96)
	**pO**_**2**_ mmHg	71.41 (± 11.85)
	**HCO**_**3**_ mmol/L	26.53 (± 2.46)
***Forced spirometry test***		
	**FVC** Liter	2.72 (± 0.83)
	**FVC** %	74.31 (± 15.91)
	**FEV**_**1**_ Liter	1.97 (± 0.67)
	**FEV**_**1**_%	65.91 (± 18.07)
	**FEV**_**1**_**/FVC** %	70.93 (± 9.39)

Demographic data of study patients (N = 35) are presented as mean(± SD) or n(%);

† AHI was measured at the time when NIV treatment was initiated

^#^ Note the normalized values of arterial blood gas as a result of the NIV treatment

BMI = body mass index; OHS = obesity hypoventilation syndrome; pCO_2_ = partial pressure of arterial carbon dioxide; pO_2_ = partial pressure of arterial oxygen; HCO_3_ = bicarbonate; FVC = forced vital capacity, FEV_1_ = forced expiratory volume in 1 s;

### Oscillometry

**Fig. 2:**
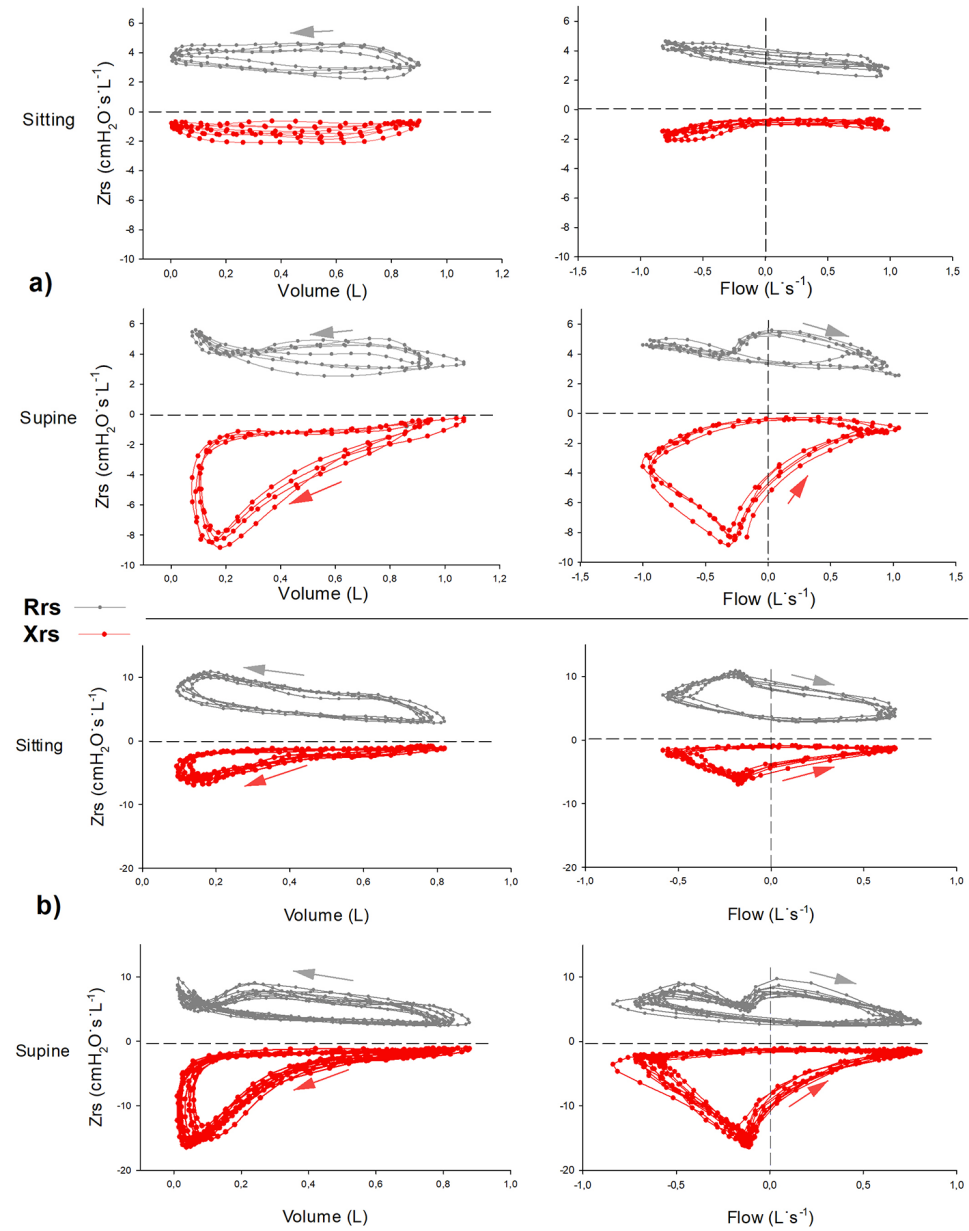
Intra-breath changes of impedance values of two representative patients with OHS. The first patient (Fig. 2a) exhibits minimal intra-breath changes in *Rrs* and *Xrs* in the sitting position, while in the supine position, a marked looping develops in the *Xrs vs V* plot. During expiration, *Xrs* departs gradually from the inspiratory path with an increasing negative slope, thus creating a clockwise loop with a significant area (*AXV*). The corresponding *Xrs vs V’* diagram reveals a gradual decrease in *Xrs* with increasing expiratory *V*’, followed by a further fall in both *Xrs* and expiratory V’ in the second half of expiration where *Xrs* approaches its lowest point with decreased *V’*. This characteristic pattern is considered the manifestation of tEFL. Inspiratory effort leads to a sharp increase in *Xrs* and, eventually, the formation of a loop. The value of the area (*AXV’*) is negative by definition due to the counter-clockwise change in *Xrs*. In connection with tEFL, a characteristic phenomenon appears in the Rrs vs. V diagram. When inspiratory activity starts, although still at a net expiratory flow, tEFL resolves, flow is abruptly restored, causing a transient increase in Rrs, likely via upper airway nonlinearities. In another subject (Fig. 2b), tEFL was already present in the sitting position with both decreased *Xrs* and *V*’ at late expiration. The change in position augmented this looping pattern of tEFL Zrs = respiratory impedance; Rrs = respiratory resistance; Xrs = respiratory reactance;

**Fig. 3:**
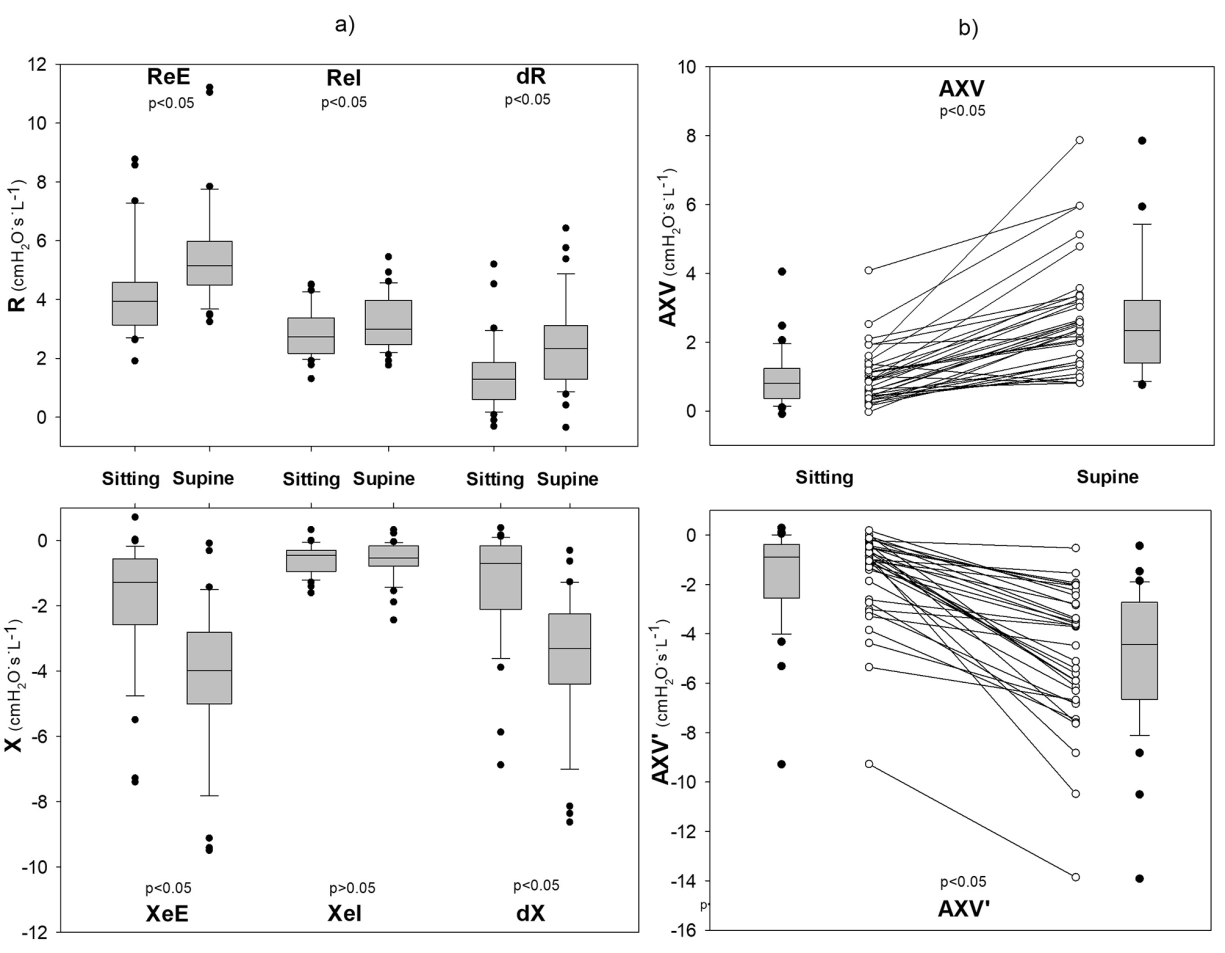
The effect of body position on the intra-breath oscillometry values. The effects of the body position on different oscillometry variables are depicted as (**a**) box-plot diagrams (ReE, ReI, ΔR, XeE, XeI, ΔX), and (**b**) individual changes and box-plot diagrams (AXV, AXV’). The supine position has a significant effect on Xrs during expiration indicating EFL during tidal breathing (tEFL). *P* values are established by the Wilcoxon signed rank test. (n = 35) ReE = end expiratory resistance; ReI = end inspiratory resistance; ΔR = difference between ReE and ReI; XeE = end expiratory reactance; XeI = end inspiratory reactance; ΔX = difference between XeE and XeI; AXV = area of Xrs vs V diagram; AXV’ = area of Xrs vs. V’ diagram;

Figure 2a and b illustrate the changes in *Zrs* in two representative patients with OHS. The change in *Xrs* loop areas between body positions (Fig. 3b) revealed notable inter-individual variability. The position dependence of the intra-breath oscillometry data is summarized in Table 3 and illustrated in Fig. 3a and b.

**Table 3:** Spirogram and intra-breath oscillometry variables of study patients

	Sitting	Supine	*p*-value
**Vt** (L)	0.92 (± 0.30)	0.95 (± 0.26)	0.10
**Frequency** (min^− 1^)	16.49 (± 4.10)	16.72 (± 4.53)	0.77
**Ti/Ttot**	0.44 (± 0.04)	0.44 (± 0.04)	0.77
**ReE** (cmH_2_O·s·L^− 1^)	4.26 (± 1.63)	5.56 (± 1.80)	< 0.001
**ReI** (cmH_2_O·s·L^− 1^)	2.81 (± 0.81)	3.37 (± 1.62)	< 0.001
**ΔR** (cmH_2_O·s·L^− 1^)	1.45 (± 1.18)	2.37 (± 1.48)	< 0.001
**ΔXmean** (cmH_2_O·s·L^− 1^)	1.19 (± 1.24)	3.33 (± 2.11)	< 0.001
**XeE** (cmH_2_O·s·L^− 1^)	-1.85 (± 1.89)	-4.17 (± 2.22)	< 0.001
**XeI** (cmH_2_O·s·L^− 1^)	-0.58 (± 0.46)	-0.61 (± 0.58)	0.71
**ΔX** (cmH_2_O·s·L^− 1^)	-1.27 (± 1.68)	-3.56 (± 2.02)	< 0.001
**AXV** (cmH_2_O)	0.93 (± 0.81)	2.53 (± 1.40)	< 0.001
**AXV’** (cmH_2_O·s·L^− 1^)	-1.52 (± 1.92)	-4.95 (± 2.89)	< 0.001
**ARV** (cmH_2_O)	-1.27 (± 0.81)	-1.46 (± 1.21)	0.34
**ARV’** (cmH_2_O·s·L^− 1^)	1.87 (± 1.26)	2.77 (± 1.83)	< 0.001

Comparison of spirogram and intra-breath oscillometry variables of study patients (N = 35) in the sitting and the supine position. Data are presented as mean(± SD); The definitions of intra-breath variables are listed in Table 1

We found tEFL (based on the presence of the *Xrs vs. V’* loop and the absolute value of AXV’) in 19 patients in the sitting position and all 35 patients in the supine position.

We observed the expected glottal interference at high CPAP values (> 5–15 H_2_Ocm) in 19 out of 33 patients (see also Supplementary Material).

All variables reflecting an expiratory decrease in *Xrs* as a potential marker of tEFL (*XeE, ΔX, AXV, AXV’*, and *ΔXmean*) changed significantly between body positions (Table 3). *ΔXmean* showed a strong correlation with *XeE* (r = 0.86), *ΔX* (r = 0.87), *AXV* (r=-0.93), and AXV’ (r=-0.95) (*p* < 0.05).

We found no correlation between oscillometry parameters indicating tEFL (*XeE, ΔX, AXV, AXV’*, and *ΔXmean*) and age, BMI, AHI, OHS stage, or spirometry parameters (*FEV1, FEV1% and FEV1/FVC*) (*p* > 0.05).

When comparing the two sexes, the oscillometry parameters reflecting tEFL were more pronounced in females compared to males in the supine position (*XeE: -5.99 vs -3.22, p < 0.001*; *ΔX: -5,11 vs -2.76, p = 0.003*; *AXV: 3.42 vs 2.07, p = 0.017 and AXV’: -6.89 vs -3.94, p = 0.006* in females and males respectively). These differences were not present in the sitting position.

When comparing subgroups with or without OSA and patients with or without severe OSA, we observed no difference in oscillometry parameters reflecting tEFL in the supine position (*XeE*, *ΔX*, *AXV, AXV’*, *p* > 0.05).

During increasing CPAP level measurements, the anticlockwise looping of AXV’ was reversible in 32 out of 33 patients. One patient showed significant looping even at a CPAP level of 20 cmH_2_O. The “optimal CPAP” (required to stop tEFL) in the study group, exhibiting significant individual differences, was 14.84 ± 4.11 cmH_2_O. The mean CPAP/EPAP used by study patients during long term treatment was 13.01 (± 2.97) cmH_2_O. We found no correlation between the defined ‘optimal’ CPAP and the actual CPAP/EPAP (*p* = 0.555).

A gradual decrease in the *Rrs* values was also observed with increasing CPAP.

**Fig. 4:**
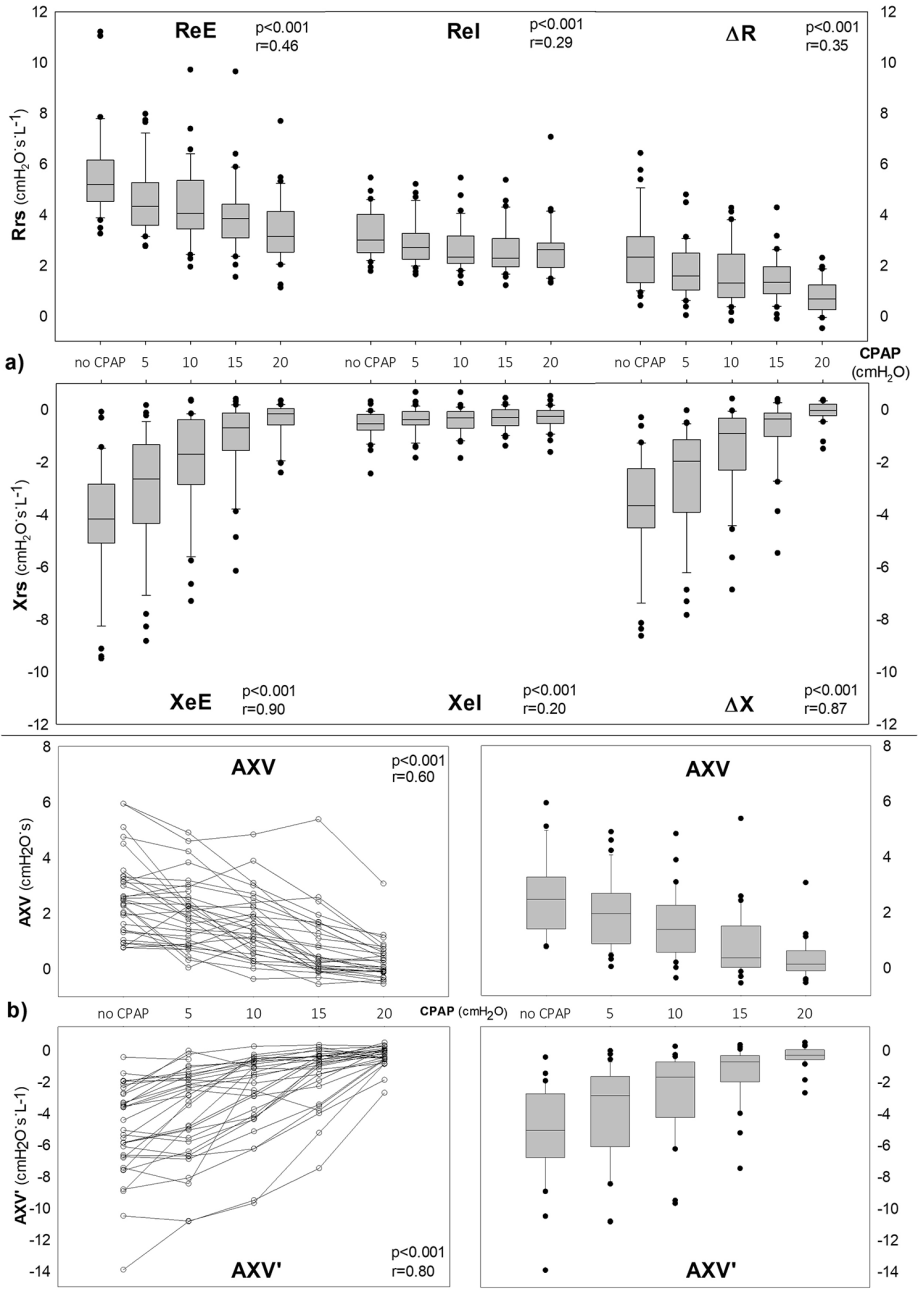
The effect of CPAP on the intra-breath oscillometry values. The effects of different CPAP levels on oscillometry variables are depicted as (**a**) box-plot diagrams (ReE, ReI, ΔR, XeE, XeI, ΔX) and (**b**) individual changes and box-plot diagrams (AXV, AXV’). With increasing CPAP levels stepwise change was visible on the Xrs during expiration indicating decreasing tEFL during tidal breathing. The CPAP level required to diminish expiratory Xrs was highly variable between patients. *P* values are established by the Friedman ANOVA test; the r values are established by Kendall’s concordance test. (n = 33) ReE = end-expiratory resistance; ReI end-inspiratory resistance; ΔR = difference between ReE and ReI; XeE = end-expiratory reactance; XeI end-inspiratory reactance; ΔX = difference between XeE and XeI; AXV = area of the Xrs vs. volume loop; AXV’= area of Xrs vs. flow loop; CPA*P* = continuous positive airway pressure;

**Fig. 5:**
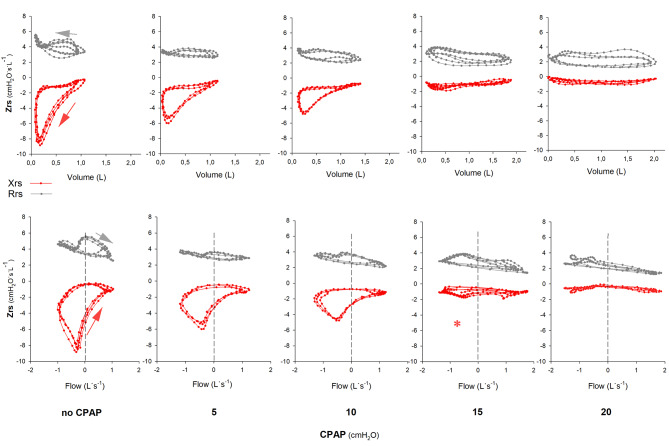
Intra-breath changes of impedance values in one representative patient with OHS. The two parts of the respiratory impedance (Zrs), the resistance (Rrs) and the reactance (Xrs) are marked in grey and red, respectively. The Rrs and Xrs data are plotted against tidal volume and tidal flow parameters. The arrows mark the direction of looping during a single breathing cycle. With a stepwise elevation of CPAP, increasing expiratory Xrs values and decreasing AXV and AXV’ loop areas indicate the gradual release from tEFL. At CPAP 10 cmH_2_O the tidal volume dependence of Xrs starts in the second half of expiration, and in this particular patient, the flow and volume dependence of Xrs was almost eliminated between CPAP 10 and 15 cmH_2_O (marked with '*'). Small gradual intra-breath changes can be seen in Rrs. The CPAP level required to reduce the flow and volume dependence of Xrs was highly variable between patients (Figure S3) Zrs = respiratory impedance; Rrs = respiratory resistance; Xrs = respiratory reactance;

Intra-breath oscillometry values during stepwise elevation CPAP are shown in Fig. 4 (see also Table S2). A representative example of a change in the looping pattern is presented in Fig. 5.

The high temporal resolution displays of CPAP measurements in all subjects revealed two types of dynamic elevations in Rrs during expiration (e.g. glottal interference), which were present in 19 of the 33 patients. One of these patterns is a trapezoid or oval loop shape in the *Rrs* vs *V* diagram reflecting steadily increasing *Rrs* during the whole expiration, whereas the other one exhibits a gradual increase in *Rrs* in the second part of expiration and sudden fall coinciding with sharp minima of *Xrs* at the expiratory limit *V*’. It is noteworthy that these elevations in expiratory *Rrs*, also reflected by the loop area *ARV*, persist during elevations in CPAP even when the clear indicators of tEFL gradually disappear in *Xrs*.

## Discussion

This study aimed to assess whether intra-breath oscillometry can be used to detect tEFL and optimize CPAP therapy in patients with OHS. We found that tEFL measured by intra-breath oscillometry is present during normal tidal breathing, becomes more pronounced in the supine position, and is reversed by CPAP in patients with OHS. Our results show that tEFL and the “optimal CPAP” value needed to diminish it (14.84 ± 4.11 cmH_2_O), are highly variable between subjects and independent of the AHI and BMI values.

tEFL is associated with tidal ventilation inhomogeneity as well as impairment of gas exchange and acts as an extra workload on the diaphragm during expiration (resulting in eccentric contraction) [24–26]. Previous studies have implied that airway function abnormalities and tEFL, in particular, are present in severely obese and OHS patients and worsen in the supine position. This has been proposed as part of the pathophysiology leading to respiratory impairment in OHS [8, 13, 27–29]. The results of our study corroborate these findings. We found that all patients with OHS present with tEFL that worsens in the supine position, although the change between the sitting and supine positions is highly variable. Additionally, we found that the newly proposed markers (*XeE, ΔX, AXV*, and *AXV’*) showed a strong correlation with the previously used tEFL marker (*ΔXmean*).

Interestingly, despite similar BMI ranges, females displayed oscillometry patterns corresponding with more intense tEFL. Given that the more intense tEFL in women is mainly present in the supine position, this phenomenon is probably independent of actual weight, although muscle mass, muscle tone or its distribution might play a role. No previous study has noted differences in tEFL in female and male obese patients, but this phenomenon might further our understanding of the development of tEFL. No previous study has noted differences in tEFL in female and male obese patients, but this phenomenon might further our understanding of the development of tEFL. Given that the differences become apparent only in the supine position, this phenomenon is probably independent of actual weight, although muscle mass, muscle tone or its distribution might play a role.

Indeed, tEFL measures were found to be independent of the severity of obesity in all OHS patients in our cohort. This seems contradictory to a previous study that found that functional residual capacity (FRC) and expiratory reserve capacity (ERV) decreased exponentially with increasing BMI in an obese population [30]. However, it is well known that not every obese patient will develop hypoventilation and our results suggest that other factors independent of BMI might contribute to increased tEFL in patients with OHS. Further studies are warranted to clarify whether the presence and level of tEFL correlates with respiratory impairment and gas exchange abnormalities in the obese population.

As anticipated, we found that the body position-related worsening of respiratory mechanics could be counteracted by CPAP [17, 24]. Reversal of tEFL could be achieved in almost all patients; however, the CPAP level required to eliminate tEFL (the “optimal CPAP”) varied between patients in our study.

The documented therapeutic effect of CPAP in OHS is thought to be the result of its ability to counteract both nocturnal upper airway collapse and small airway closure during tidal breathing [7, 12, 31]. Coexisting OSA is frequent in patients with OHS, but airway patency issues and reduction of lung volumes are not directly related, and OHS may appear without morbid obesity (BMI < 40 kg·m^− 2^) or a high AHI [32]. This is further supported by the fact that we found no correlation between AHI and BMI or between AHI and awake supine oscillometry variables reflecting tEFL. Moreover, subgroup analysis did not show an association between OSA severity (AHI ≥ 5/h or AHI ≥ 30/h) or BMI and awake supine oscillometry variables reflecting tEFL. Despite this, CPAP therapy is usually titrated based on airway patency during sleep studies in OHS, and its effect on absolute lung volume is not monitored. While airway patency is required for maintaining ventilation during sleep, CPAP levels based on this may be insufficient to restore EELV, adequately improve ΔV/ΔQ, and unload respiratory muscles overloaded in OHS. Studies establishing CPAP as a viable treatment for OHS used settings acquired during AHI based CPAP titration, with mean values ranging from 10 to 15 cmH_2_O [33–36]. As a noteworthy finding in our study, these CPAP levels were not sufficient to eliminate tEFL in all OHS patients in our cohort. Additionally, ‘optimal’ CPAP values based on oscillometry measurements did not correlate with the settings used by study patients during their long term ventilation therapy. Transcutaneous CO_2_ monitoring during therapy titration might provide more reliable values for effective CPAP, although whether this results in clinically improved outcomes remains to be seen [37]. Our results suggest that intra-breath oscillometry measurements provide important additional information for optimizing CPAP treatment in patients with OHS. This is in line with recent findings suggesting that CPAP may be helpful even in patients without severe OSA [11]. Further studies are needed to determine whether CPAP levels based on awake supine intra-breath oscillometry variables are effective in achieving clinical goals and improving long-term outcomes in patients with OHS.

Glottal interference, previously described in animal models, was present in close to 60% of patients with OHS in our study during CPAP measurements [21]. These patients exhibited increased Rrs during expiration with similar kinetics to voluntary glottal narrowing [38]. As it has been previously noted and reinforced by our results here, resistance fluctuations in the upper airway can appear parallel with intrapulmonary and small airway mechanical changes [38]. We found that the values of expiratory Rrs, ARV and ARV’ typically persist during increasing CPAP settings, meanwhile Xrs variables improve gradually with stepwise elevation of CPAP. Therefore, XeE, ΔX, AXV and AXV’ appear to be reliable indicators of the presence of tEFL during CPAP measurements. Further research is warranted to analyse the mechanical effect of glottal activity on small airway mechanics via elevation of intrabronchial pressure; however, characterisation of this phenomenon was not the aim of this study. Visualisation of the dynamic change in Rrs during expiration with intra-breath mapping allows clear distinction between glottal origin and other possible causes. It is also important to note that glottal interference might explain intolerance of high initial airway pressures and stresses the need for gradual stepwise increase in CPAP values both during titration and long-term therapy to increase adherence.

Our study had some limitations. tEFL was assumed to be the result of reduced lung volume in our study; however, ERV and FRC were not measured, as this would have required plethysmography impractical in the supine position. Patients with a possible obstructive pathophysiology were excluded to rule out other contributors to tEFL. Forced expiratory spirometry did not identify any significant obstruction in the study group. Additionally, oscillometry detection of tEFL may be hindered by glottal interference, as resistance fluctuations in the upper airway can appear parallel to intrapulmonary and small airway mechanical changes [38]. To eliminate the effects of upper airway obstruction, we performed a study in awake patients. To identify the glottal narrowing potentially accompanying higher levels of PEEP previously described in animal models [21], we used a visual depiction of the respiratory *Xrs* vs. *V’* and *V* relationships and tEFL markers that more accurately assess the different patterns of dynamic shifts in *Xrs* and *Rrs* during tidal breathing. We identified possible glottal narrowing accompanying CPAP measurements in 19 of 33 patients; however, further studies are needed to verify which tEFL markers can accurately identify this phenomenon.

Finally, the current study used a stepwise elevation of CPAP measurements with quite large jumps in order to quickly and efficiently distinguish between levels of tEFL. The stepwise application of CPAP is important to avoid hyperinflation related bias and patient discomfort, however a more precise titration of CPAP could yield more optimal CPAP settings. Further studies are needed to identify the ideal CPAP titration protocol during oscillometry measurements and polysomnography verification of oscillometry results for a clinically feasible, precise diagnostic algorithm.

In the context of oscillometry employed in a number of clinical scenarios, the general application of this 30–60 min test during OHS assessment seems feasible and might optimize treatment in a disease where long-term survival is still poor [39, 40]. However, implementation of our technique in clinical studies is hindered by the fact that the currently available commercial oscillometry devices do not operate at elevated airway pressures.

## Conclusions

OHS, as a leading cause of chronic respiratory failure, requires a clear understanding of its pathophysiology and the ways to optimize treatment. Our results demonstrate that intra-breath oscillometry can provide important information about position-related tEFL in patients with OHS and may aid in optimizing CPAP therapy.

### Electronic supplementary material

Below is the link to the electronic supplementary material.

Supplementary Material 1

## Data Availability

The datasets generated and analysed during the current validation study are available from the corresponding author on reasonable request.

## List of Abbreviations

AHI: Apnoea-hypopnea index
BMI: Body mass index
COPD: Chronic obstructive pulmonary disease
CPAP: Continuous positive airway pressure
EELV: End-expiratory lung volume
EFL: Expiratory flow limitation
FEV_1_: Forced expiratory volume in 1s
FVC: Forced vital capacity
NEP: Negative expiratory pressure
OHS: Obesity hypoventilation syndrome
OSA: Obstructive sleep apnoea
P: Pressure
PEEP: Positive end expiratory pressure
Rrs: Respiratory resistance
tEFL: Tidal expiratory flow limitation
V: Volume
V’: Flow
WOB: Work of breathing
Xrs: Respiratory reactance
Zrs: Respiratory impedance
