# Supplementary material for: Using intra-breath oscillometry in obesity hypoventilation syndrome to detect tidal expiratory flow limitation: a potential marker to optimize CPAP therapy
Source: BMC Pulm Med. 2023 Nov 28;23:477. doi: 10.1186/s12890-023-02777-x (PMC10685591; doi:10.1186/s12890-023-02777-x)
Supplement: Supplementary file 1 — Supplementary Material 1 [file 12890_2023_2777_MOESM1_ESM.docx]

**Supplementary material**

**Using intra-breath oscillometry in obesity hypoventilation syndrome to detect tidal expiratory flow limitation: a potential marker to optimize CPAP therapy**

Szabolcs Baglyas^1^, Luca Valkó^1^, Vivien Móró^1^, Eszter Podmaniczky^1^, Dorottya Czövek^2^, Gergely Makan^3^, Zoltán Gingl^3^, János Gál^1^, Zoltán Hantos^1†^, András Lorx^1†^

^1^ Department of Anesthesiology and Intensive Therapy, Semmelweis University, Budapest, Hungary

^2^ Department of Pediatrics, Semmelweis University, Budapest, Hungary

^3^ Department of Technical Informatics, University of Szeged, Szeged, Hungary

† Shared last authorship

Corresponding author:

Szabolcs Baglyas

Department of Anesthesiology and Intensive Therapy

Semmelweis University, 1082 Üllői út 78/B, Budapest, Hungary

baglyas.szabolcs@semmelweis.hu

| **Table S1**: Diagnostic criteria of study patients (n = 35). Data was collected at the time long-term respiratory support was initiated  Y = yes; N = no; md = missing data; NA = non applicable; M = male; F = female | | | | | | | | | | | | | |
| --- | --- | --- | --- | --- | --- | --- | --- | --- | --- | --- | --- | --- | --- |
|  | Daytime hypercapnia  (pCO_2_>45 mmHg) | HCO_3_^-^>27 (mmol/l) | Epworth sleepiness scale | Signs of right heart failure | Worsening hypercapnia during sleep (>10 mmHg) decrease) | Age (year) | Male / Female | Cardial comorbidity | Weight (kg) | Height (cm) | BMI (kg/m^2^) | OHS staging | Baseline AHI (/hour) |
| Patient_1 | Y | Y | 10 | Y | NA | 78 | M | Y | 99 | 175 | 32.327 | 4 | 83 |
| Patient_2 | Y | Y | 4 | Y | NA | 57 | M | N | 125.0 | 162 | 47.63 | 3 | md |
| Patient_3 | Y | Y | md | Y | NA | 47 | M | Y | 295.0 | 180 | 91.049 | 4 | 1.2 |
| Patient_4 | Y | Y | 17 | Y | NA | 65 | M | N | 137.0 | 170 | 47.405 | 3 | md |
| Patient_5 | Y | Y | 7 | N | NA | 63 | M | Y | 193.2 | 182 | 58.326 | 4 | 18.5 |
| Patient_6 | N | N | 20 | Y | Y | 52 | M | N | 176.5 | 178 | 55.706 | 3 | 47.4 |
| Patient_7 | Y | Y | 15 | Y | NA | 53 | M | N | 193 | 170 | 66.782 | 3 | 13.9 |
| Patient_8 | Y | Y | 15 | Y | NA | 71 | M | Y | 152.5 | 173 | 50.954 | 4 | 6.8 |
| Patient_9 | Y | Y | 10 | Y | NA | 46 | F | N | 106 | 152 | 45.88 | 3 | 10.4 |
| Patient_10 | N | N | 15 | N | Y | 57 | M | N | 134.5 | 176 | 43.421 | 1 | 20.6 |
| Patient_11 | Y | Y | 8 | Y | NA | 61 | F | N | 118 | 158 | 47.268 | 1 | 35.8 |
| Patient_12 | Y | Y | 16 | Y | NA | 55 | M | Y | 142.2 | 172 | 48.067 | 4 | 98.4 |
| Patient_13 | Y | Y | 10 | N | NA | 48 | M | Y | 161.4 | 178 | 50.941 | 4 | 2.0 |
| Patient_14 | N | N | 8 | N | Y | 47 | M | N | 130 | 176 | 41.968 | 1 | 14.5 |
| Patient_15 | Y | Y | 10 | N | NA | 67 | M | N | 125 | 170 | 43.253 | 3 | 20.7 |
| Patient_16 | Y | N | 8 | Y | NA | 57 | F | N | 112.4 | 170 | 38.893 | 3 | md |
| Patient_17 | Y | N | 0 | N | NA | 39 | F | N | 127.4 | 154 | 53.719 | 3 | 41.6 |
| Patient_18 | Y | Y | 5 | N | NA | 51 | F | N | 165 | 175 | 53.878 | 3 | 7.1 |
| Patient_19 | Y | Y | 8 | Y | NA | 52 | M | N | 135 | 170 | 46.713 | 3 | 11.9 |
| Patient_20 | Y | Y | 22 | N | NA | 50 | F | N | 130 | 155 | 54.11 | 3 | 10.2 |
| Patient_21 | Y | Y | 4 | N | NA | 48 | F | N | 128 | 170 | 44.291 | 3 | 3.6 |
| Patient_22 | Y | Y | 24 | N | NA | 61 | F | N | 140 | 156 | 57.528 | 3 | 22.3 |
| Patient_23 | Y | Y | 14 | Y | NA | 61 | M | N | 118 | 183 | 35.235 | 3 | 8.1 |
| Patient_24 | Y | Y | 7 | Y | Y | 54 | M | N | 227 | 187 | 64.915 | 3 | 4.7 |
| Patient_25 | Y | Y | 20 | N | NA | 39 | M | N | 139 | 176 | 44.873 | 3 | 2.5 |
| Patient_26 | Y | Y | 19 | Y | NA | 57 | M | N | 139.4 | 172 | 47.12 | 3 | 97.3 |
| Patient_27 | N | N | 10 | N | Y | 60 | M | N | 123 | 178 | 38.821 | 2 | 23 |
| Patient_28 | Y | Y | 1 | Y | NA | 68 | F | Y | 91.5 | 167 | 32.809 | 4 | 26.4 |
| Patient_29 | N | Y | 8 | N | Y | 59 | M | N | 116 | 170 | 40.138 | 3 | 8.4 |
| Patient_30 | Y | Y | 2 | N | NA | 46 | M | N | 141.1 | 174 | 46.605 | 3 | 77.1 |
| Patient_31 | Y | Y | 8 | Y | NA | 56 | M | Y | 186.2 | 185 | 54.405 | 4 | 3.5 |
| Patient_32 | Y | Y | 13 | N | Y | 61 | F | Y | 147.2 | 155 | 61.27 | 4 | md |
| Patient_33 | Y | Y | 10 | N | NA | 60 | F | Y | 119 | 164 | 44.244 | 4 | 1.7 |
| Patient_34 | Y | Y | 4 | Y | NA | 66 | F | Y | 109.4 | 153 | 46.734 | 4 | 8 |
| Patient_35 | Y | Y | 7 | Y | NA | 59 | M | Y | 135 | 165 | 49.587 | 4 | 11.4 |

| **Figure S1.** Schematics of the oscillometry setup. Antibacterial/viral filter and mouthpiece assembly: PBF-100 (Piston Medical Inc. Budapest. Hungary). Pressure (P) and airflow (V’) sensors: Honeywell model 26PCAFA6D (Golden Valley. MN. USA). Breathing tube and non-invasive respiratory device (A40; Philips Respironics. Murrysville. PA. USA) were alternately connected to the setup via a T-tap for measurements for “no CPAP” and at CPAP≥5 cmH_2_O. respectively. The shunt tube allowed for equilibration of static pressures between the front and back chambers of the loudspeaker-in-box system. |
| --- |
| 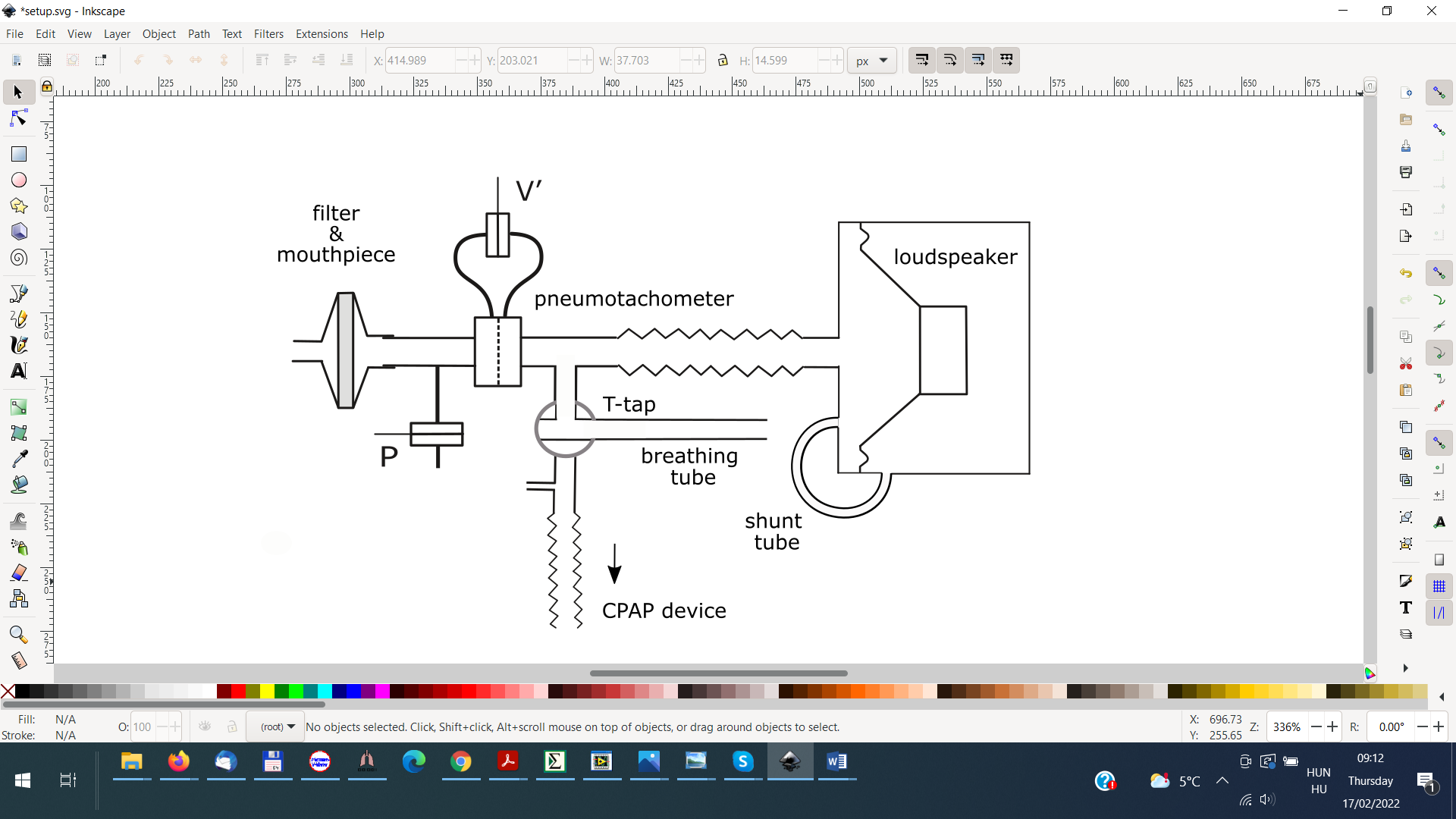 |

| **Table S2:** Extended list of measured and calculated values of intra-breath oscillometry | | |
| --- | --- | --- |
| **Parameter** | **Unit** | Definition |
| **Vt** | **Litre** | Tidal volume |
| **Frequency** | **min^-1^** | Breathing frequency |
| **Ti/Ttot** |  | Inspiratory time divided by breathing cycle time |
| **Te/Ttot** |  | Expiratory time divided by breathing cycle time |
| **Rmean** | **cmH_2_O·s·L^-1^** | Mean respiratory resistance during the breathing cycle |
| **RmeanE** | **cmH_2_O·s·L^-1^** | Mean respiratory resistance during the expiration |
| **RmeanI** | **cmH_2_O·s·L^-1^** | Mean respiratory resistance during the inspiration |
| **ReE** | **cmH_2_O·s·L^-1^** | Respiratory resistance at the end of expiration |
| **ReI** | **cmH_2_O·s·L^-1^** | Respiratory resistance at the end of inspiration |
| **ΔR** | **cmH_2_O·s·L^-1^** | Difference between ReE and ReI |
| **ARV** | **cmH_2_O·s** | Area of the resistance vs volume diagram |
| **ARV’** | **cmH_2_O·s·L^-1^** | Area of the resistance vs flow diagram |
| **Xmean** | **cmH_2_O·s·L^-1^** | Mean respiratory reactance during the breathing cycle |
| **XmeanE** | **cmH_2_O·s·L^-1^** | Mean respiratory reactance during the expiration |
| **XmeanI** | **cmH_2_O·s·L^-1^** | Mean respiratory reactance during the inspiration |
| **EFL index (ΔXmean)** | **cmH_2_O·s·L^-1^** | Difference between *XmeanE* and *XmeanI* |
| **XeE** | **cmH_2_O·s·L^-1^** | Respiratory reactance at the end of expiration |
| **XeI** | **cmH_2_O·s·L^-1^** | Respiratory reactance at the end of inspiration |
| **ΔX** | **cmH_2_O·s·L^-1^** | Difference between XeE and XeI |
| **AXV** | **cmH_2_O·s** | Area of the reactance vs volume diagram |
| **AXV’** | **cmH_2_O** | Area of the reactance vs flow diagram |

| **Table S3**: Oscillometry values corresponding to different CPAP levels. Data are presented as mean±SD.  Vt = tidal volume; Ti = time of the inspiration; Ttot = time of the breathing cycle; ReE = end expiratory resistance; ReI = end inspiratory resistance; ΔR = difference between ReE and ReI; ΔXmean = difference between the mean expiratory and mean inspiratory reactance; XeE = end expiratory reactance. XeI = end inspiratory reactance. ΔX = difference between XeE and XeI. AXV = Area of Xrs vs volume diagram; AXV’= Area of Xrs vs flow diagram; | | | | | | | | | | | | |
| --- | --- | --- | --- | --- | --- | --- | --- | --- | --- | --- | --- | --- |
| **Mean. SD** | **No CPAP**  **N = 33** | | **CPAP 5 cmH2O**  **N = 33** | | **CPAP 10 cmH2O**  **N = 31** | | **CPAP 15 cmH2O**  **N = 29** | | **CPAP 20 cmH2O**  **N = 29** | | **Friedman**  **p-value** | **r** |
| **Vt**  **(liter)** | 0.96 | ±0.25 | 1.10 | ±0.37 | 1.21 | ±0.40 | 1.24 | ±0.47 | 1.17 | ±0.44 | 0.0000 | 0.2045 |
| **Frequency**  **(min^-1^)** | 16.70 | ±4.31 | 15.47 | ±4.05 | 15.31 | ±4.11 | 15.70 | ±3.99 | 16.68 | ±4.29 | 0.0045 | 0.1065 |
| **Ti/Ttot** | 0.44 | ±0.04 | 0.43 | ±0.04 | 0.42 | ±0.04 | 0.41 | ±0.03 | 0.42 | ±0.05 | 0.0438 | 0.0558 |
| **ReE**  **(cmH_2_O*s*L^-1^)** | 5.65 | ±1.82 | 4.64 | ±1.38 | 4.35 | ±1.62 | 4.08 | ±1.59 | 3.43 | ±1.35 | 0.0000 | 0.4561 |
| **ReI**  **(cmH_2_O*s*L^-1^)** | 3.21 | ±0.92 | 2.83 | ±0.89 | 2.67 | ±0.93 | 2.60 | ±0.96 | 2.64 | ±1.15 | 0.0000 | 0.2860 |
| **ΔR**  **(cmH_2_O*s*L^-1^)** | 2.44 | ±1.45 | 1.81 | ±1.09 | 1.69 | ±1.18 | 1.47 | ±0.95 | 0.80 | ±0.80 | 0.0000 | 0.3467 |
| **ΔXmean**  **(cmH_2_O*s*L^-1^)** | -3.40 | ±2.15 | -2.32 | ±1.79 | -1.60 | ±1.51 | -0.81 | ±1.15 | -0.26 | ±0.56 | 0.0000 | 0.7906 |
| **XeE**  **(cmH_2_O*s*L^-1^)** | -4.25 | ±2.26 | -3.11 | ±2.32 | -2.06 | ±2.04 | -1.23 | ±1.57 | -0.42 | ±0.71 | 0.0000 | 0.9046 |
| **XeI**  **(cmH_2_O*s*L^-1^)** | -0.58 | ±0.54 | -0.41 | ±0.51 | -0.40 | ±0.51 | -0.36 | ±0.41 | -0.30 | ±0.45 | 0.0000 | 0.1971 |
| **ΔX**  **(cmH_2_O*s*L^-1^)** | -3.67 | ±2.04 | -2.69 | ±2.04 | -1.65 | ±1.76 | -0.87 | ±1.33 | -0.12 | ±0.43 | 0.0000 | 0.8672 |
| **AXV**  **(cmH_2_O)** | 2.58 | ±1.42 | 1.98 | ±1.24 | 1.53 | ±1.18 | 0.84 | ±1.20 | 0.34 | ±0.70 | 0.0000 | 0.6063 |
| **AXV’ (cmH_2_O*s*L^-1^)** | -5.09 | ±2.92 | -3.95 | ±2.93 | -2.70 | ±2.57 | -1.50 | ±1.83 | -0.40 | ±0.65 | 0.0000 | 0.7966 |
| **ARV**  **(cmH_2_O)** | -1.55 | ±1.30 | -1.82 | ±1.31 | -2.42 | ±1.17 | -2.38 | ±1.55 | -2.01 | ±1.55 | 0.00201 | 0.1242 |
| **ARV’**  **(cmH_2_O·s·L^-1^)** | 3.10 | ±1.89 | 2.78 | ±1.84 | 2.68 | ±2.85 | 2.53 | ±1.41 | 1.61 | ±1.51 | 0.00358 | 0.1117 |

| **Figure S2**: Variables of intra-breath oscillometry depicted on *Zrs vs V* and the *Zrs vs V’* diagram.. The two parts of the respiratory impedance (*Zrs*). the resistance (*Rrs*) and the reactance (*Xrs*). are marked as grey and red colours respectively. The *Rrs* and *Xrs* recordings of a single patient are mapped against tidal volume and tidal flow parameters. The arrows mark the direction of looping during a single breathing cycle.  Zrs = respiratory impedance; Rrs = respiratory resistance; Xrs = respiratory reactance; ReE = end expiratory resistance; ReI= end inspiratory resistance; ΔR = difference between ReE and ReI; XeE = end expiratory reactance; XeI = end inspiratory reactance; ΔX = difference between XeE and XeI; AXV = area of Xrs vs V diagram; AXV’ = area of Xrs vs V’ diagram; ARV = area of Rrs vs V diagram; ARV’ = area of Rrs vs V’ diagram; |
| --- |
| 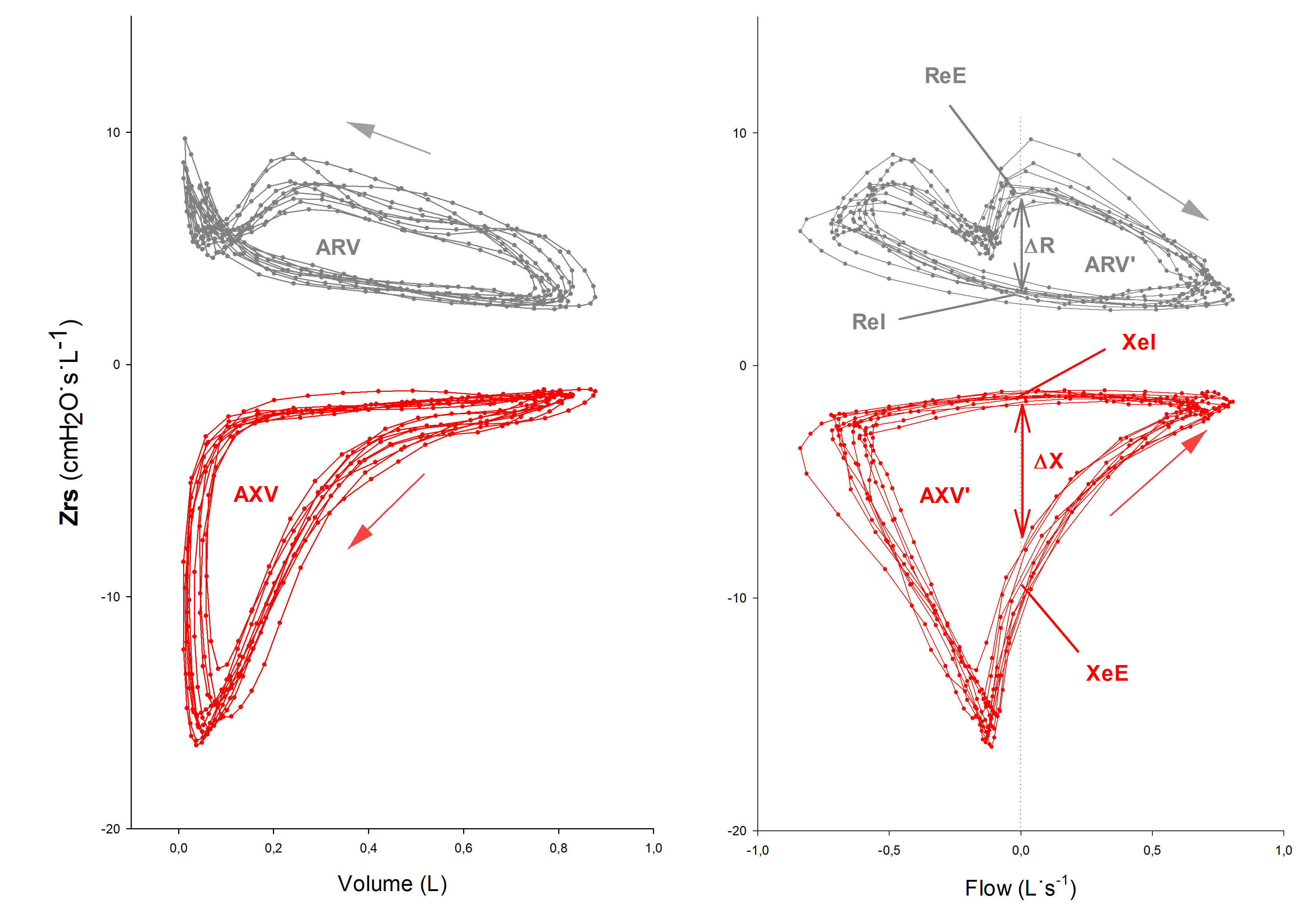 |

| **Figure S3:** Intra-breath changes of impedance values of the study patients during different CPAP levels.  The two components of respiratory impedance (*Zrs*). resistance (*Rrs*) and reactance (*Xrs*) are marked with grey and red colours respectively. The *Rrs* and *Xrs* recordings of a single patient are mapped against tidal volume and tidal flow parameters. The arrows mark the direction of looping during a single breathing cycle.  The high temporal resolution displays of CPAP measurements in all subjects revealed two types of dynamic elevations in Rrs during expiration, which were present in 19 of the 33 patients. One of these patterns is a trapezoid or oval loop shape in the Rrs vs V diagram reflecting steadily increasing Rrs during the whole expiration, whereas the other one exhibits a gradual increase in Rrs towards the end of expiration. It is noteworthy that these elevations in expiratory Rrs, also reflected by the loop area ARV, persist during elevations in CPAP even when the clear indicators of tEFL gradually disappear in Xrs.  As an important finding of our study, visual depiction of the respiratory impedance vs. flow and volume are also helpful in recognizing different patterns of dynamic shifts in Xrs and Rrs during tidal breathing, indicative of glottal narrowing during CPAP measurements. Glottal interference, previously described in animal models, was present in close to 60 % of patients with OHS in our study during CPAP measurements[1]. These patients exhibited increased Rrs during expiration with similar kinetics to voluntary glottal narrowing[2]. As it has been previously noted and reinforced by our results here, resistance fluctuations in the upper airway can appear parallel with intrapulmonary and small airway mechanical changes[2]. We found that the values of expiratory Rrs, ARV and ARV’ typically persist during increasing CPAP settings, meanwhile Xrs variables improve gradually with stepwise elevation of CPAP. Therefore, XeE, ΔX, AXV and AXV’ appear to be reliable indicators of the presence of tEFL during CPAP measurements. Further research is warranted to analyse the mechanical effect of glottal activity on small airway mechanics via elevation of intrabronchial pressure; however, characterisation of this phenomenon was not the aim of this study. Visualisation of the dynamic change in Rrs during expiration with intra-breath mapping allows clear distinction between glottal origin and other possible causes. It is also important to note that glottal interference might explain intolerance of high initial airway pressures and stresses the need for gradual stepwise increase in CPAP values both during titration and long-term therapy to increase adherence.  Two types of expiratory interference of the glottis can be visible: one resembling a “trapeze-like” looping pattern in the *Rrs vs volume* diagram with continuously increased *Rrs* during expiration (*ac). ad). ah). ai). al). aq). ar). at). au). av). aw). az). ba)*). or another with a gradual end-expiratory increase in *Rrs* (*ae). af). aj). am). bc). bf)*).  Zrs=respiratory impedance. Rrs=respiratory resistance. Xrs= respiratory reactance |
| --- |
| 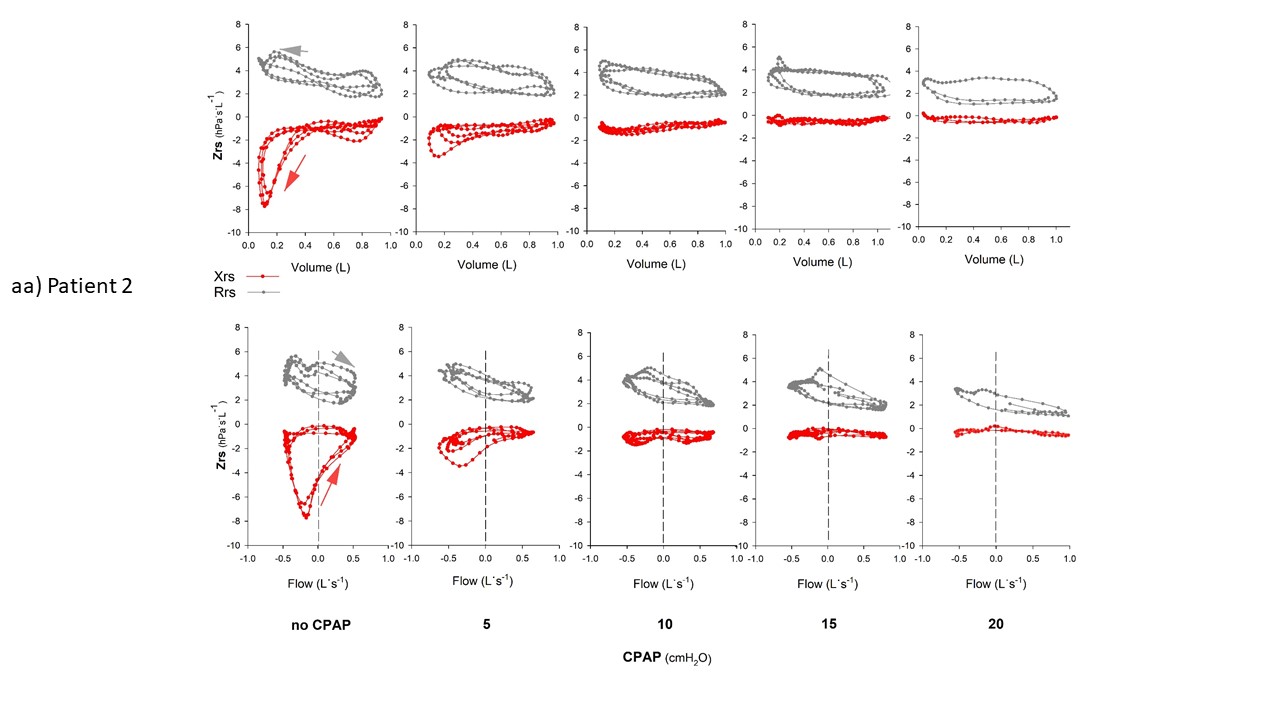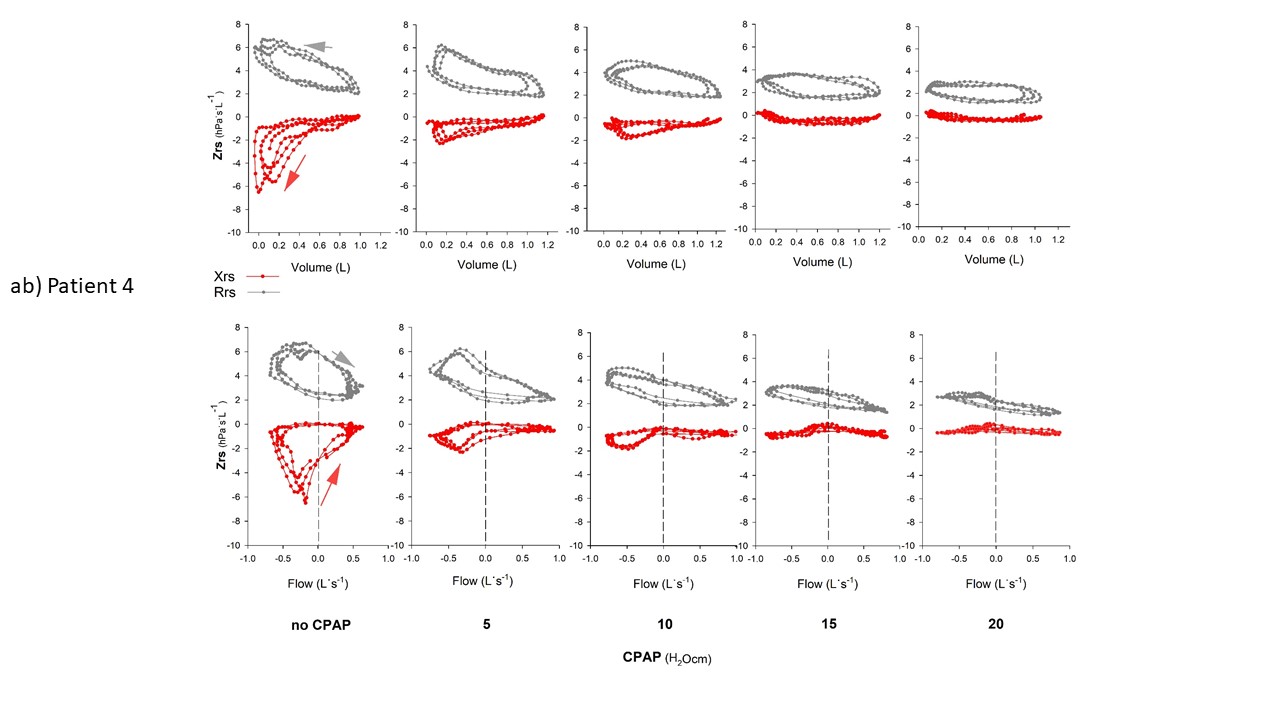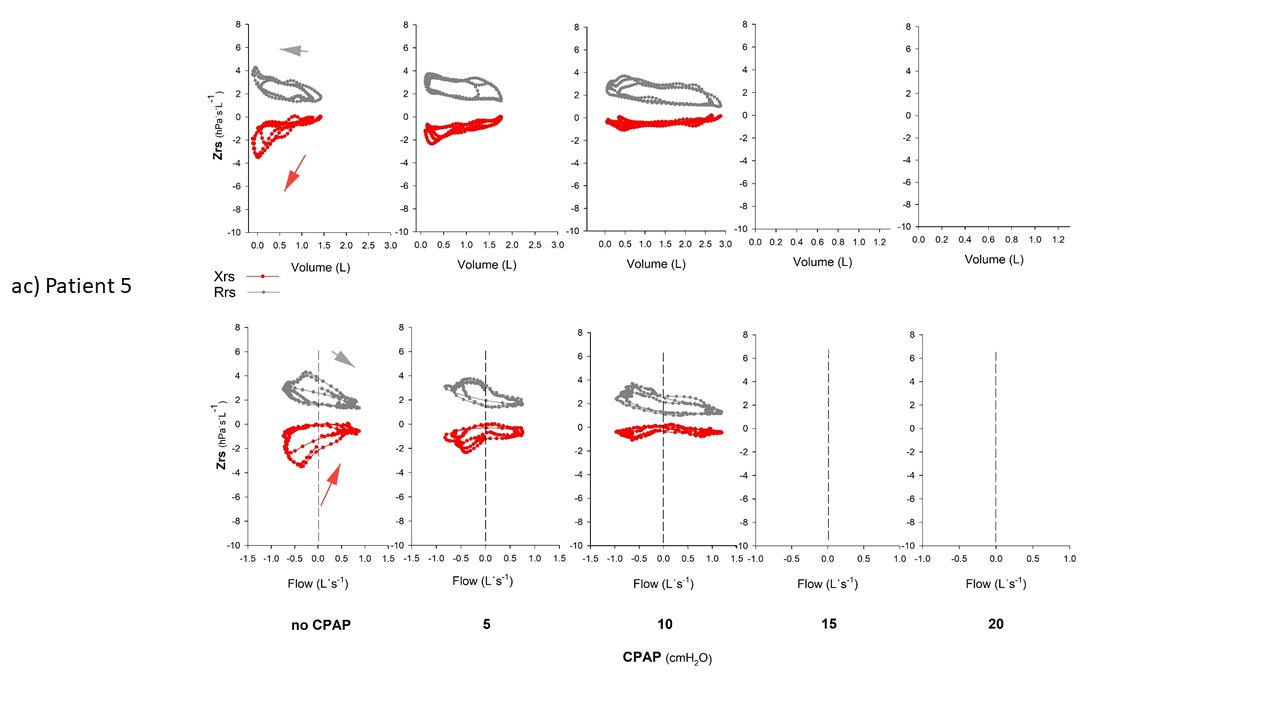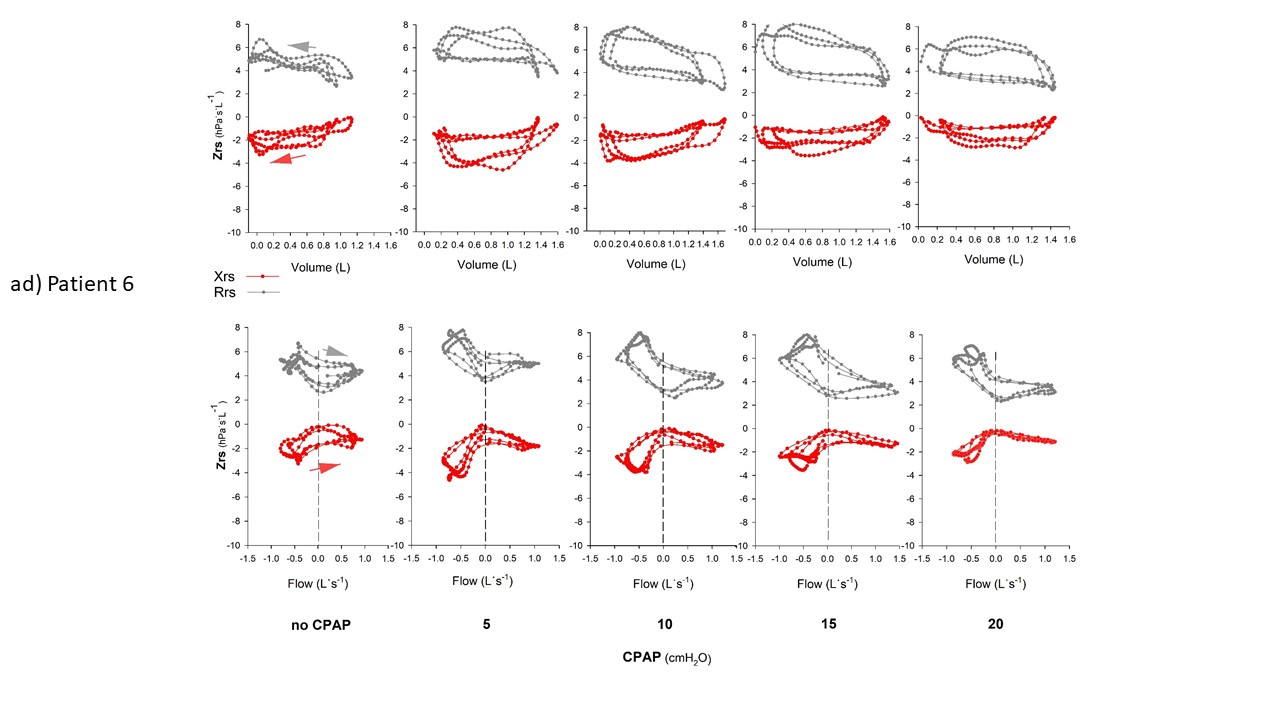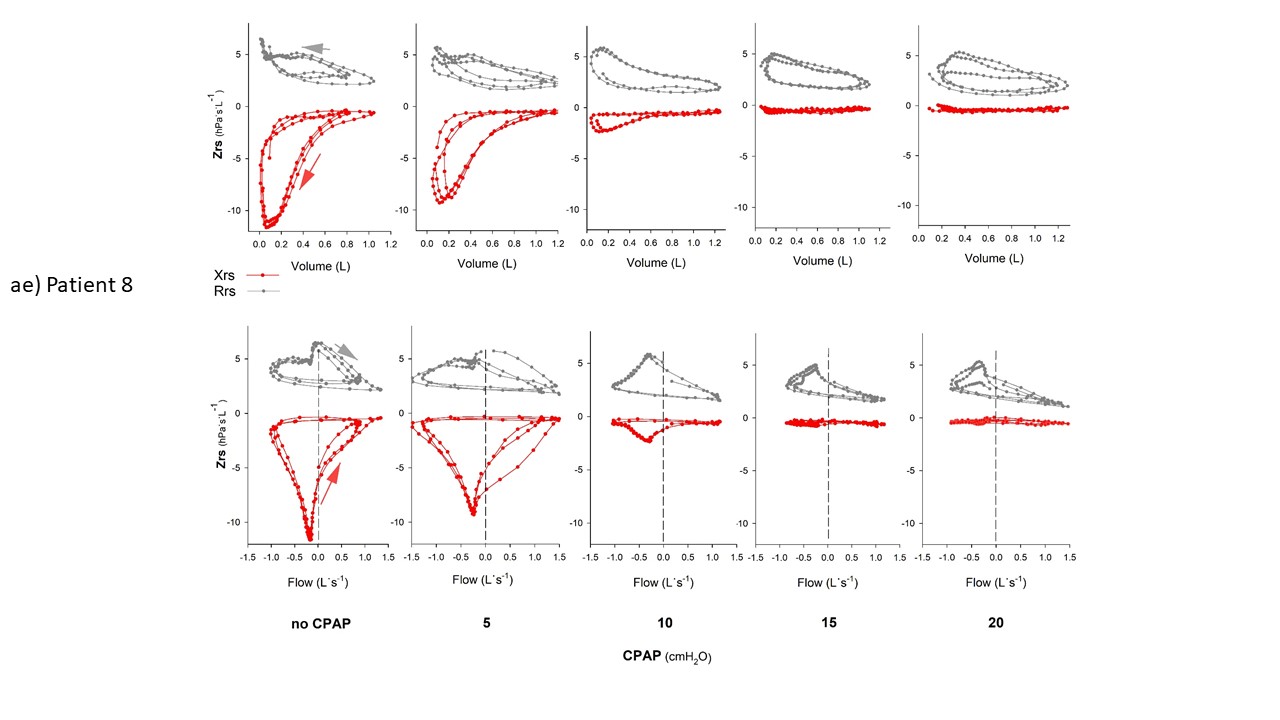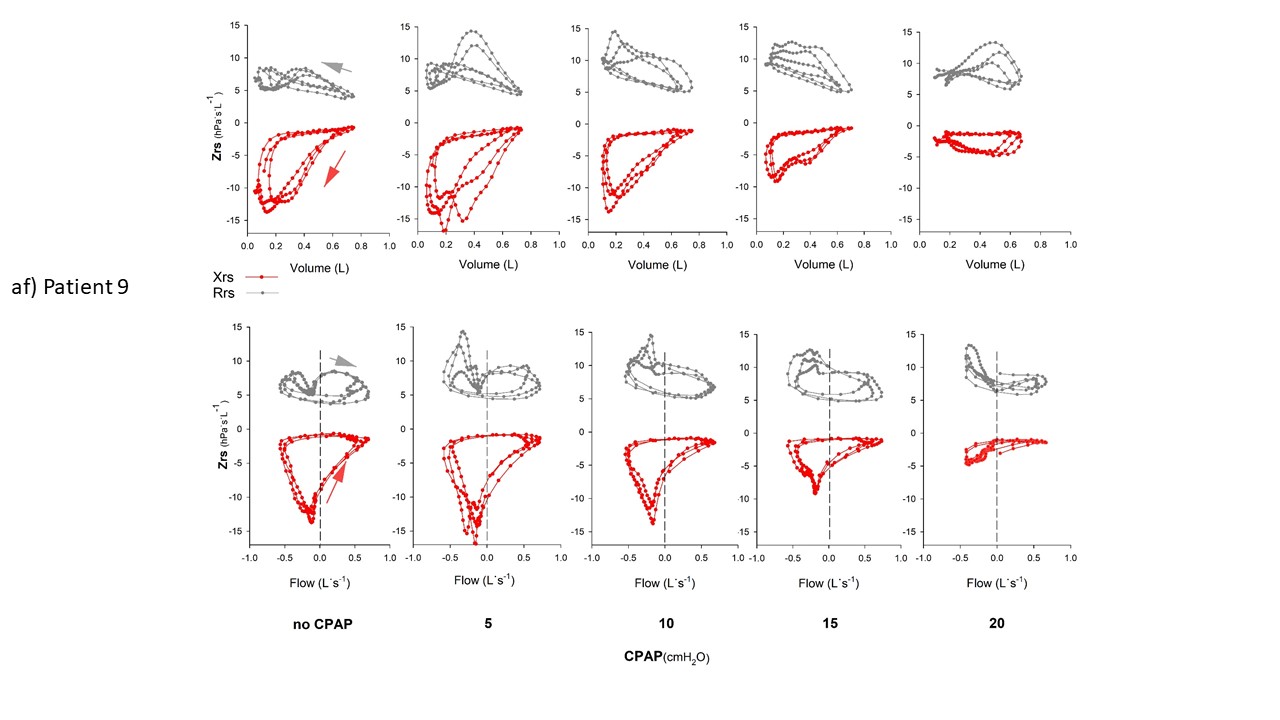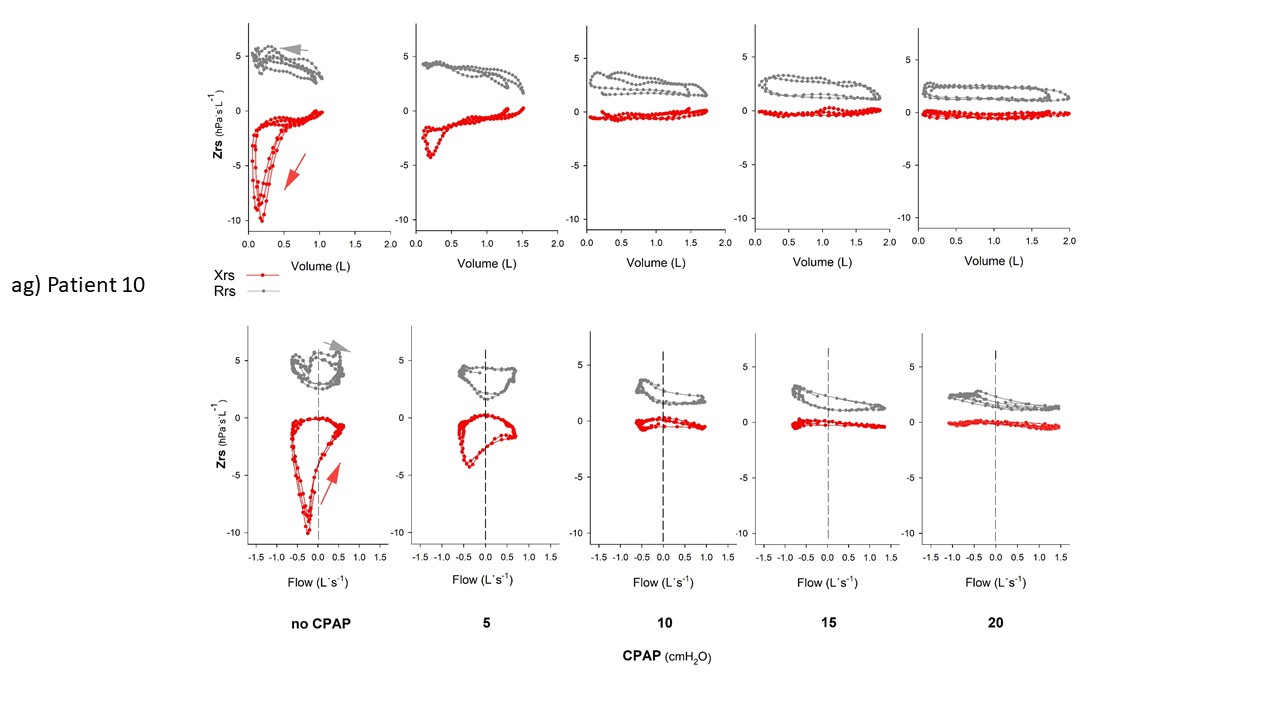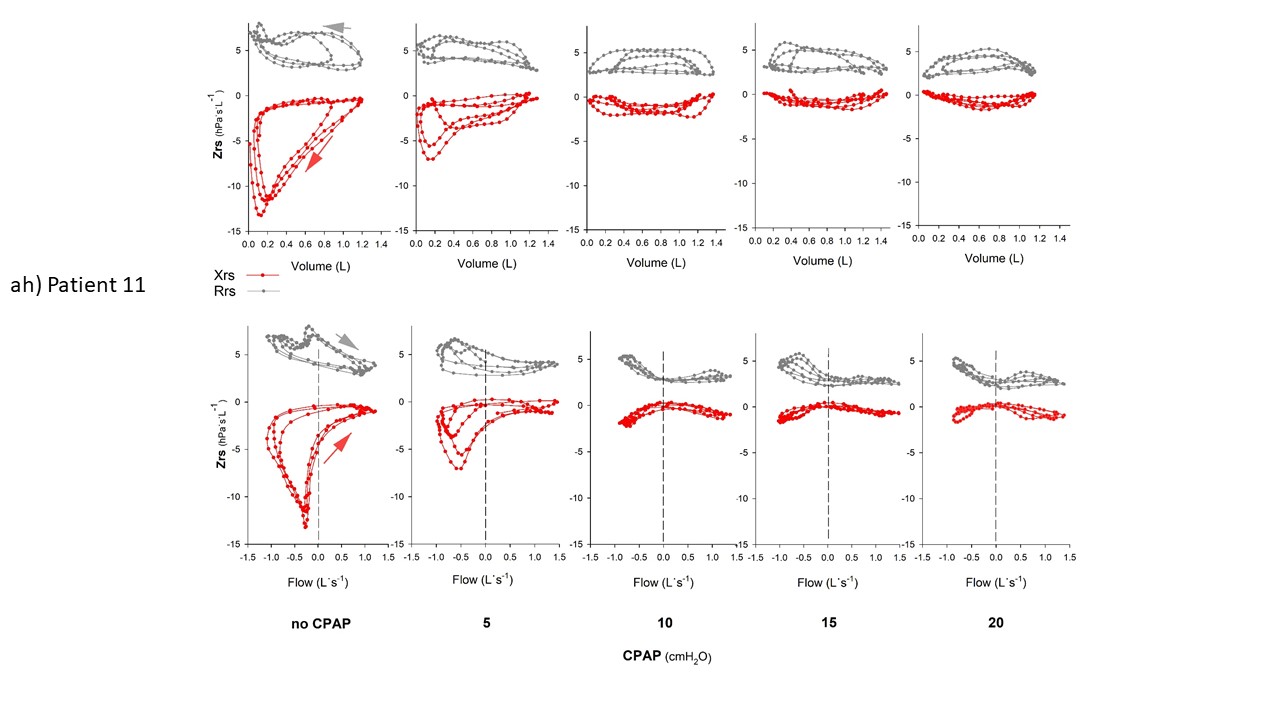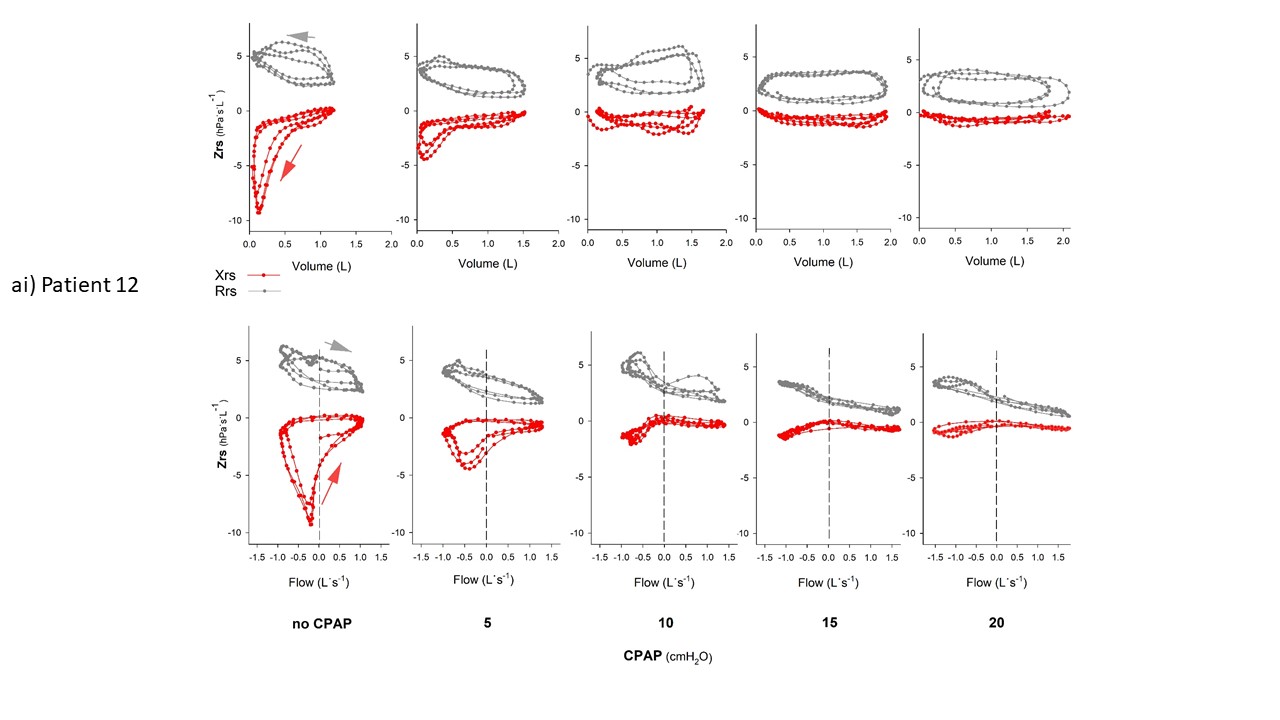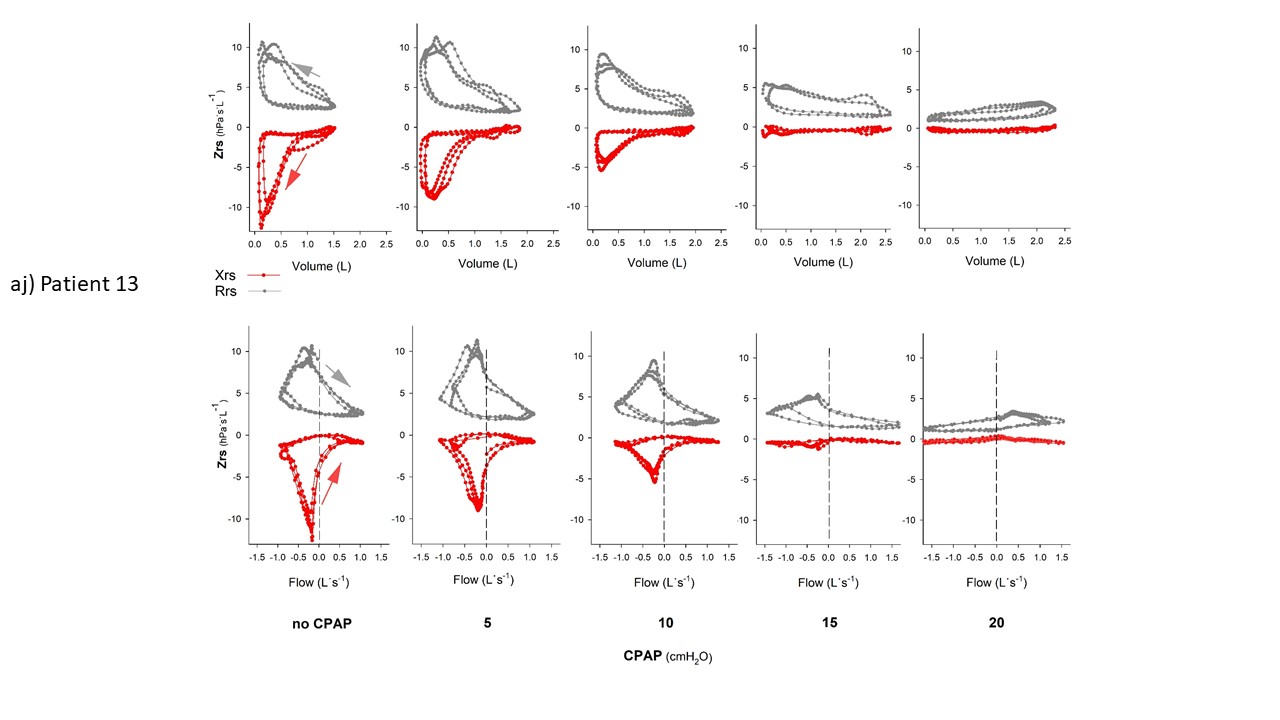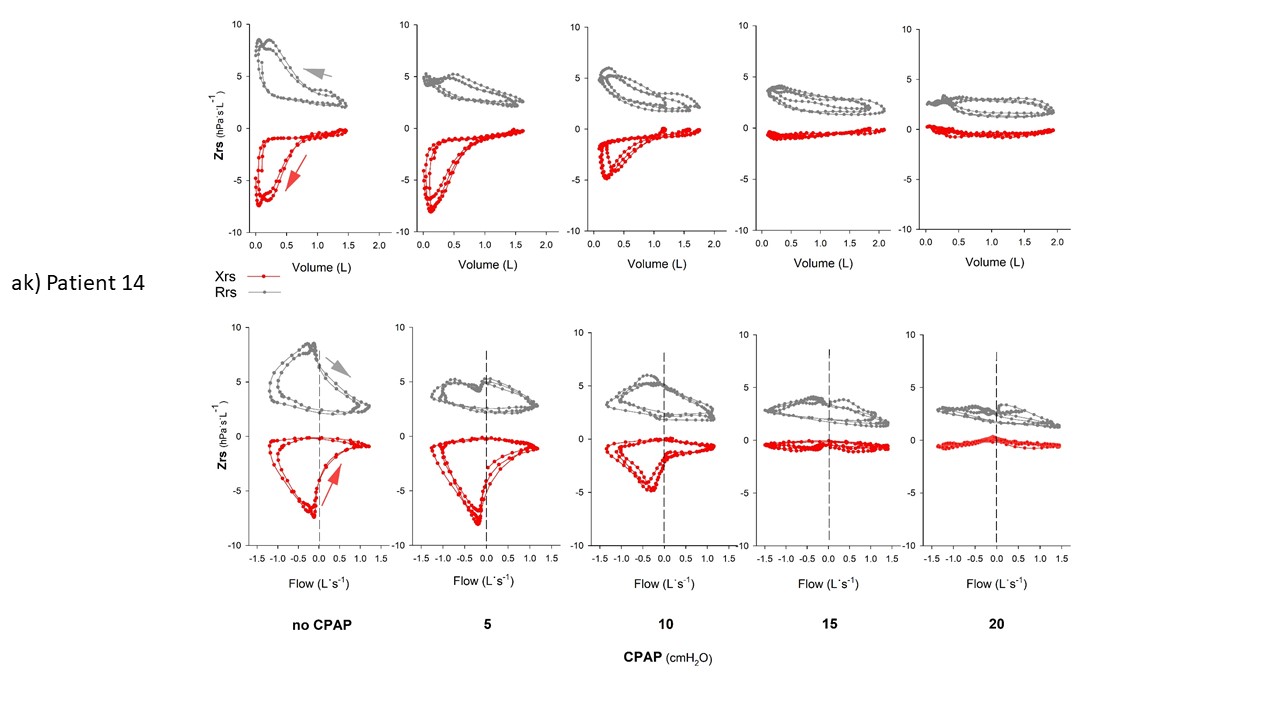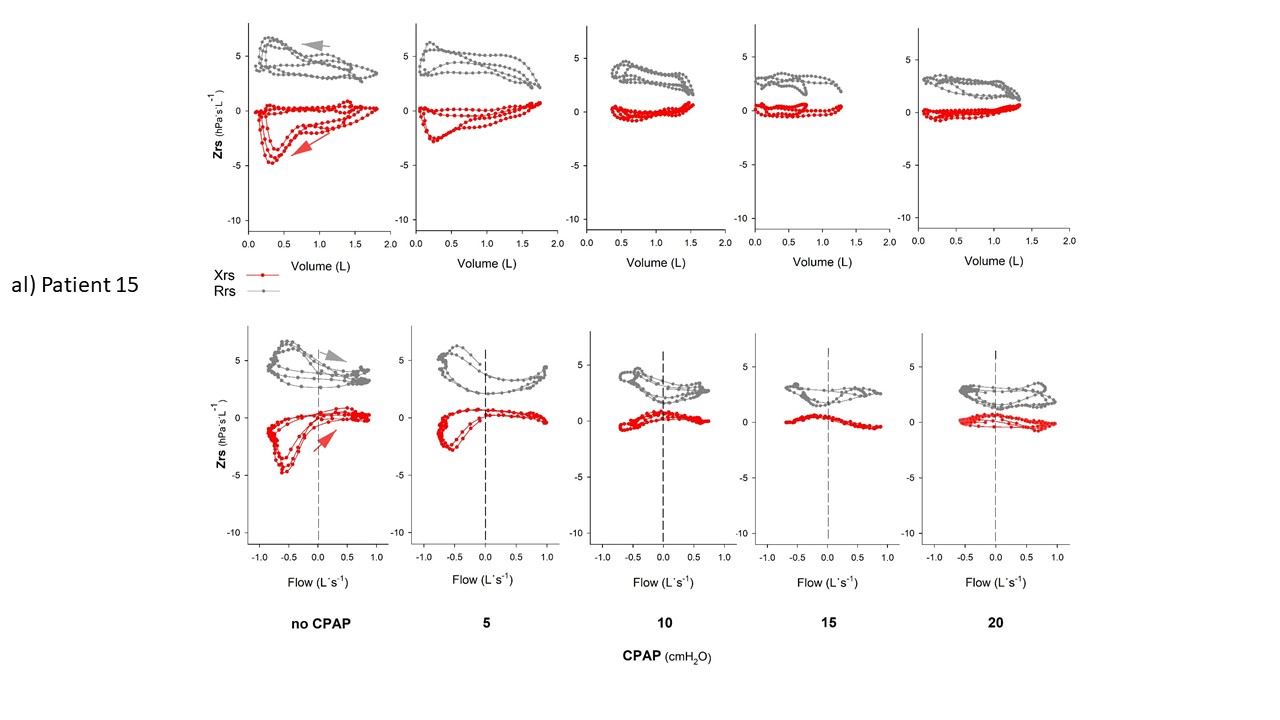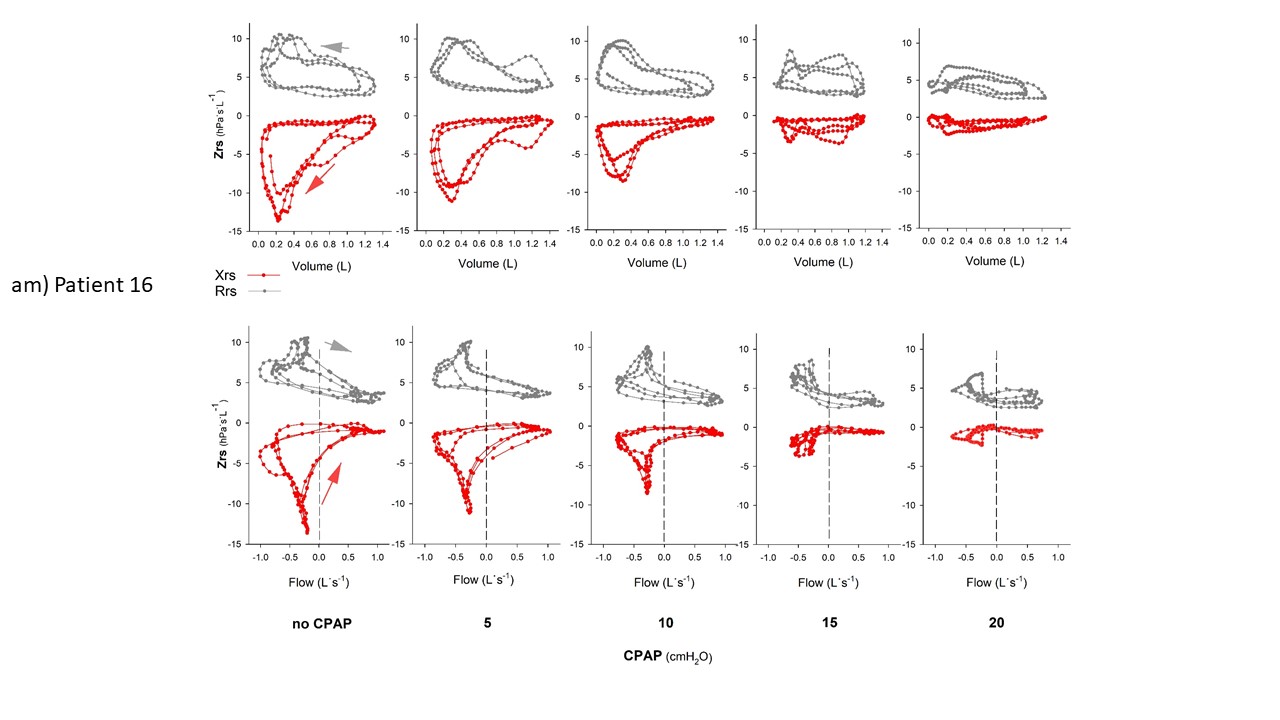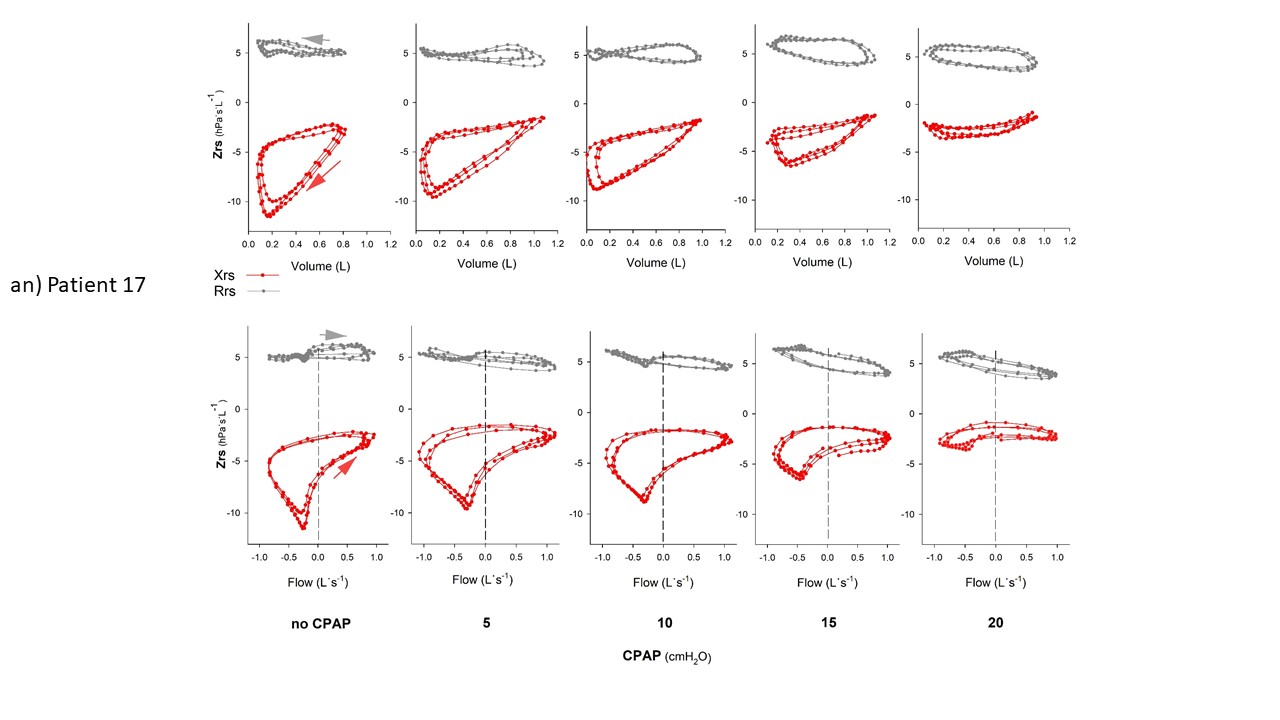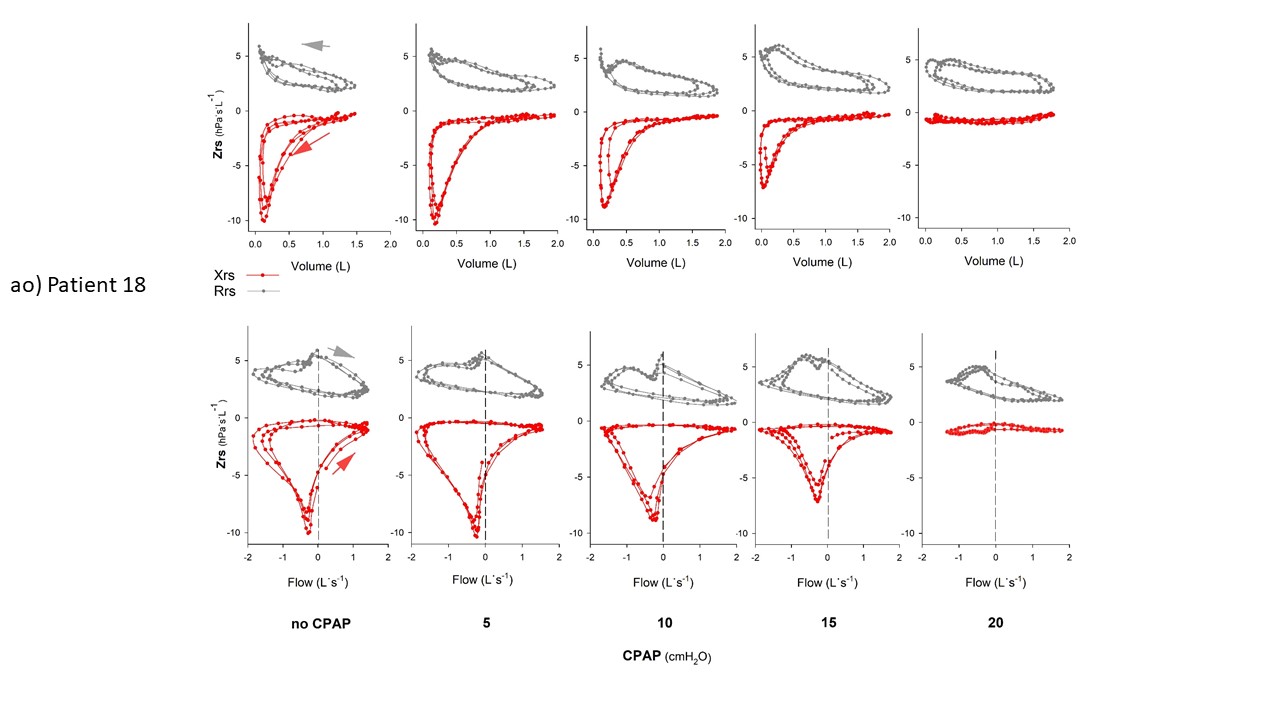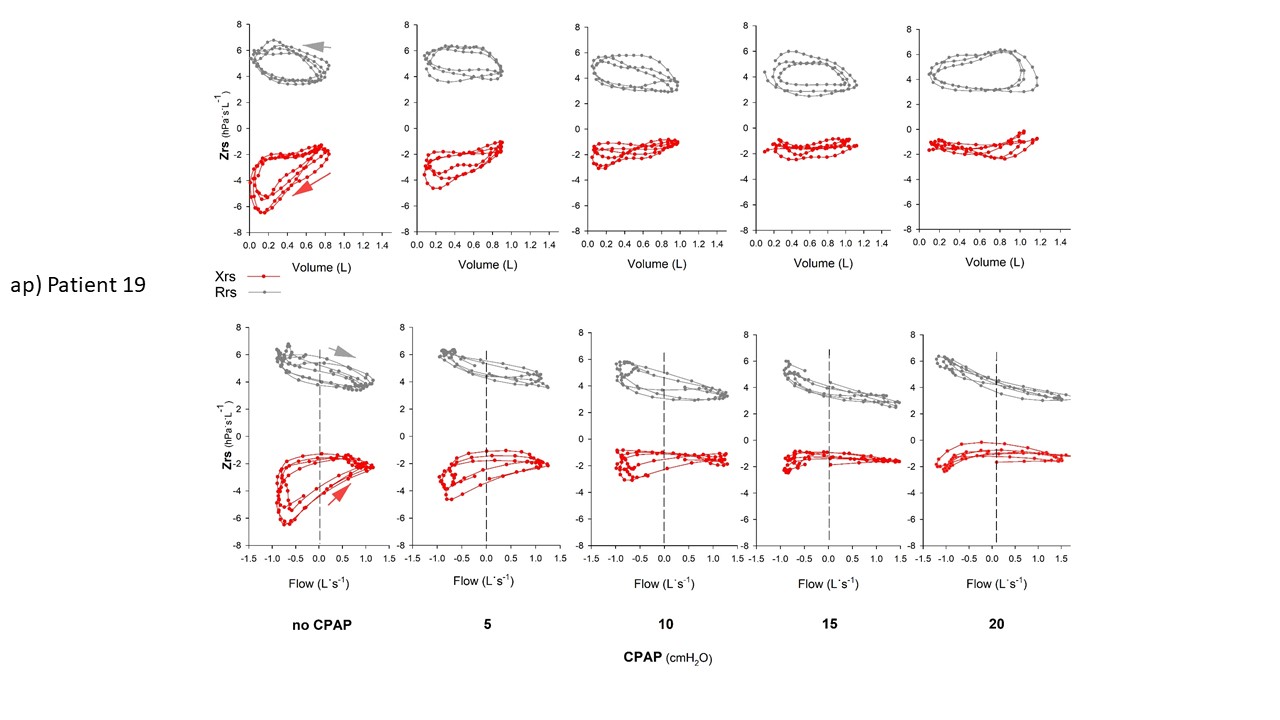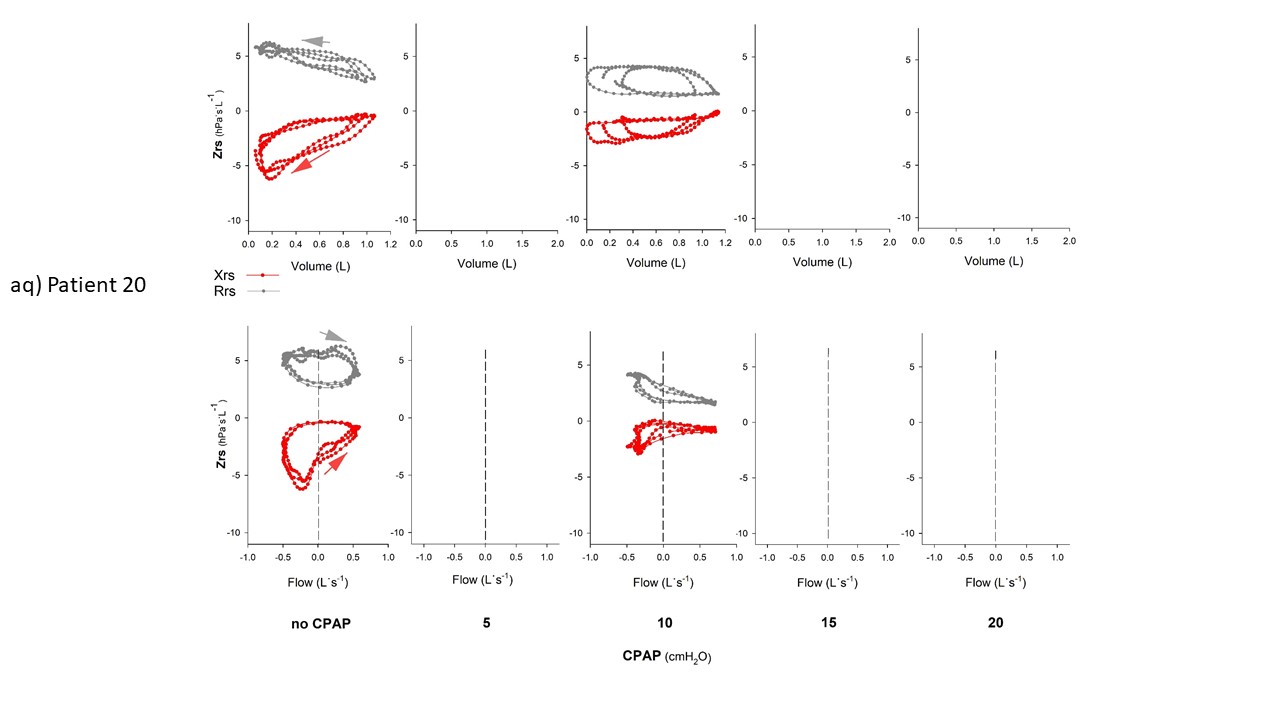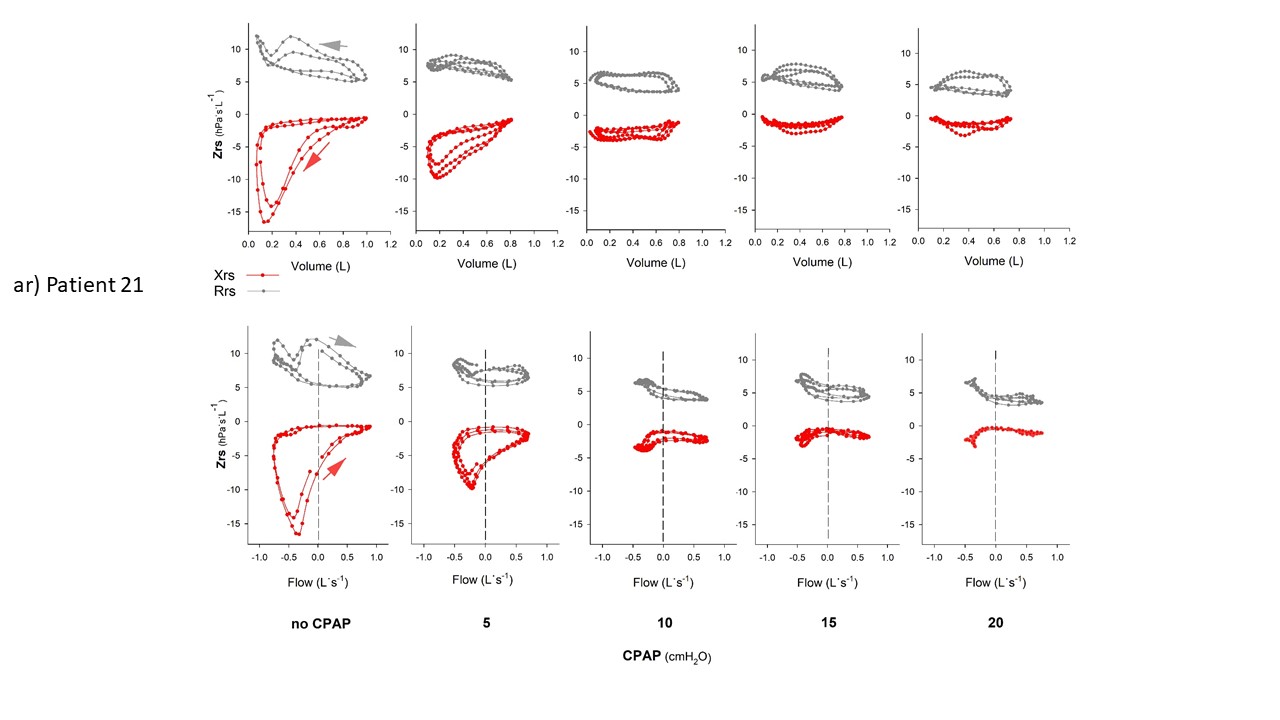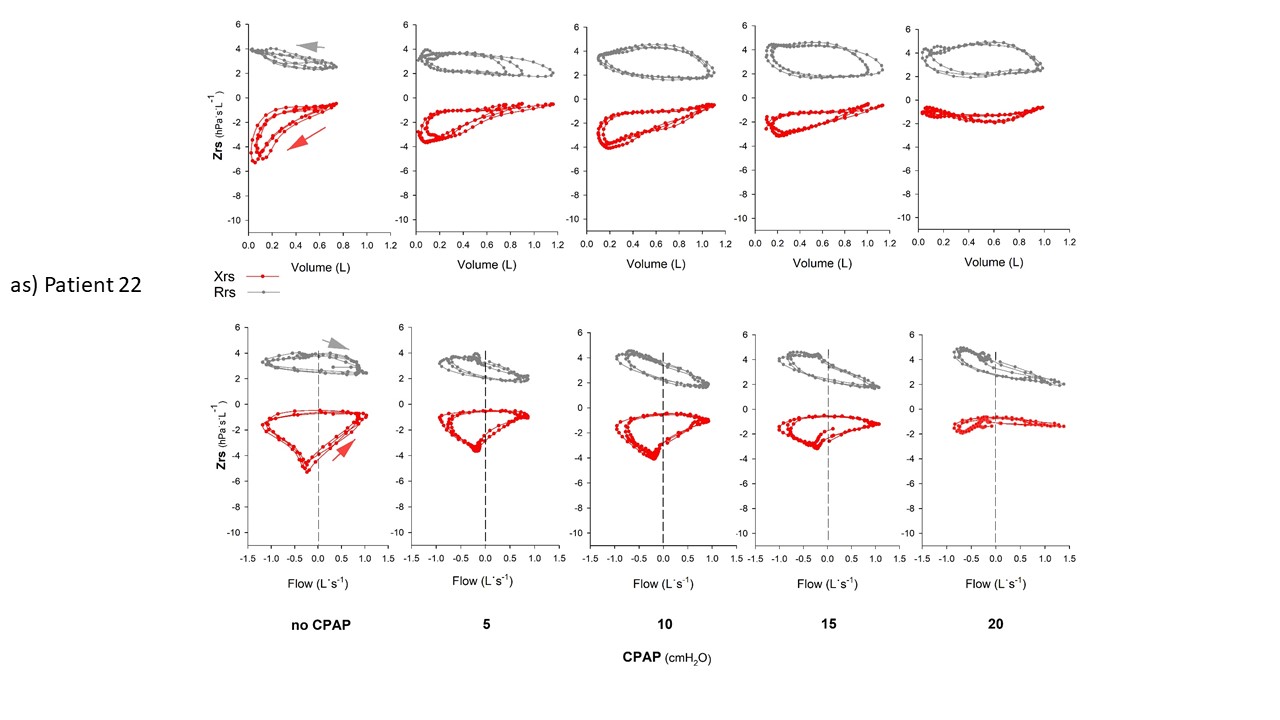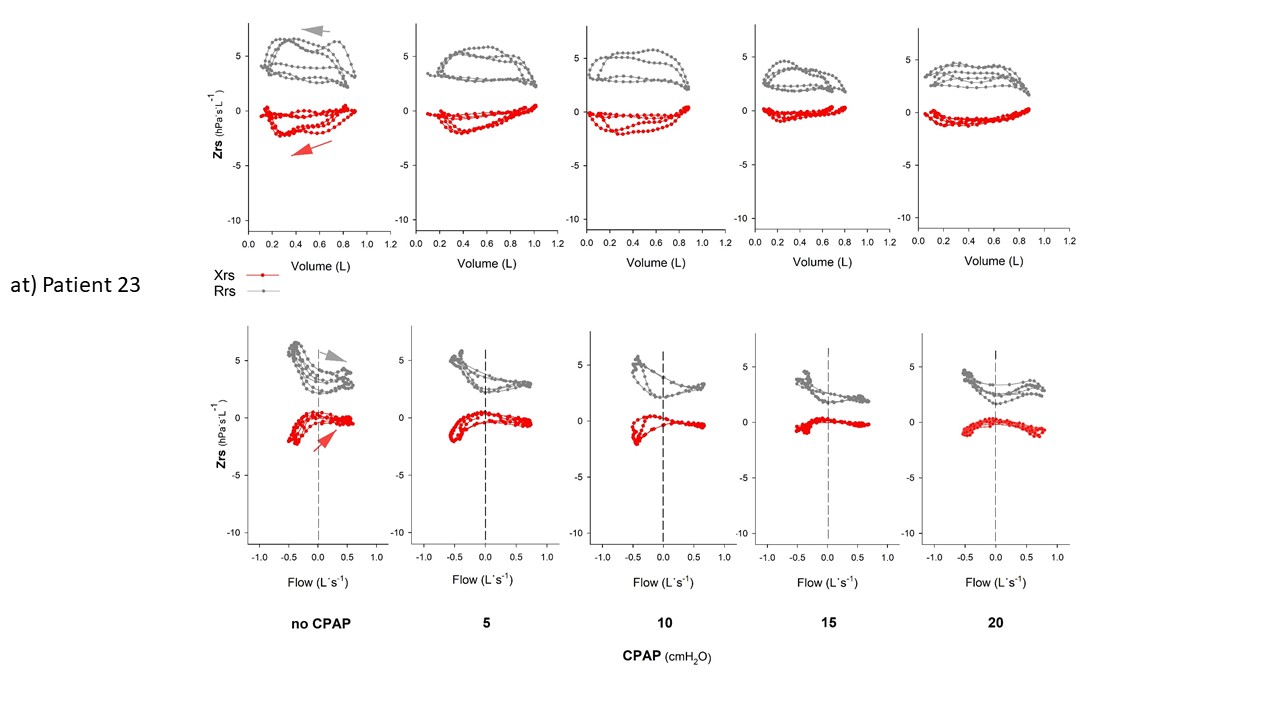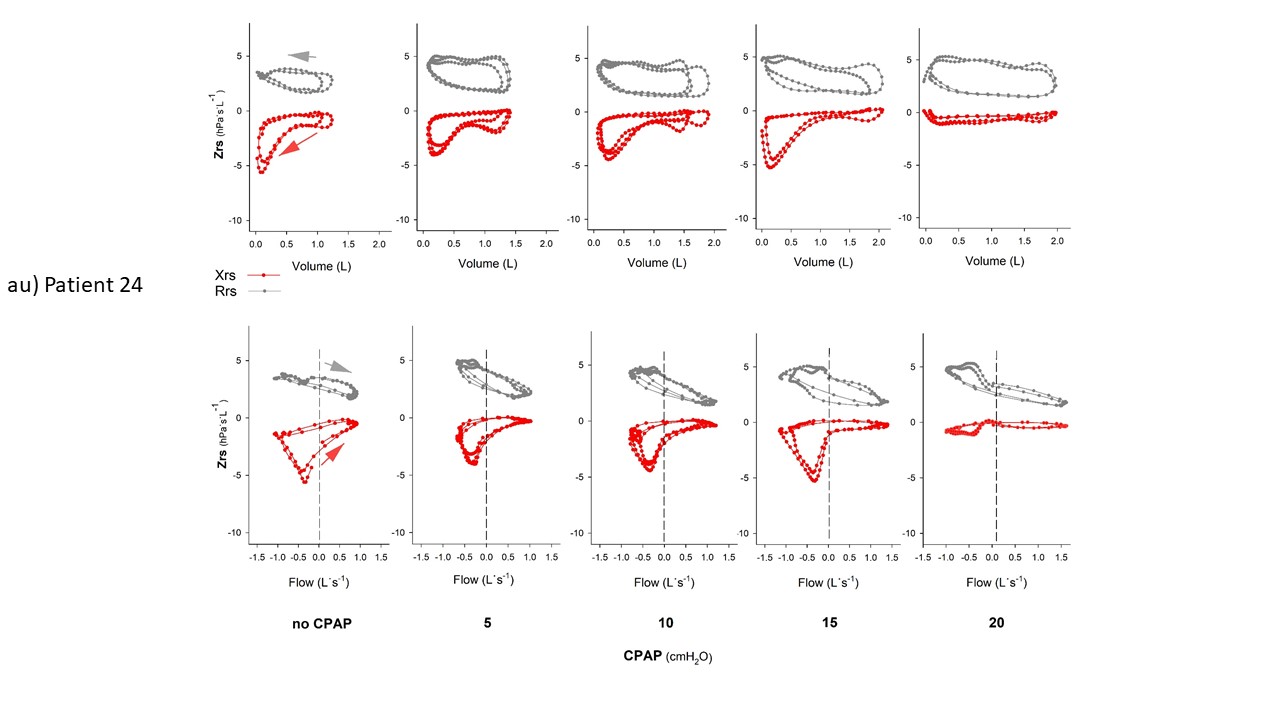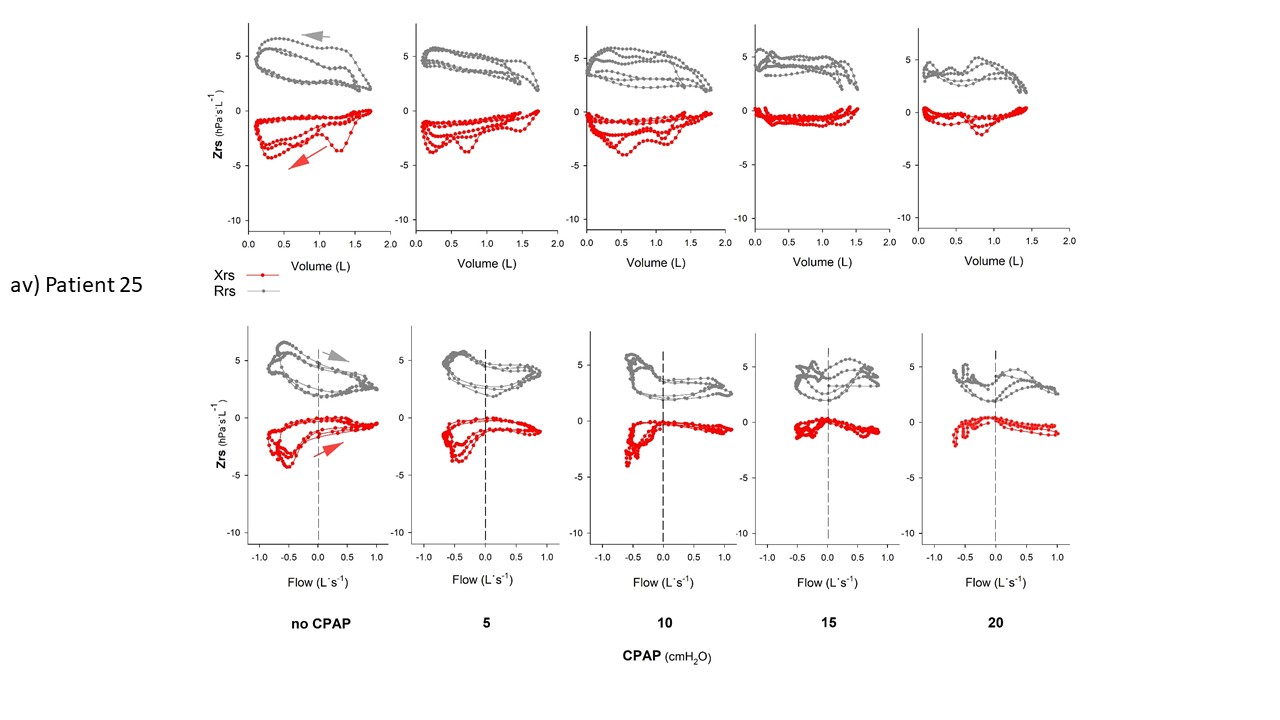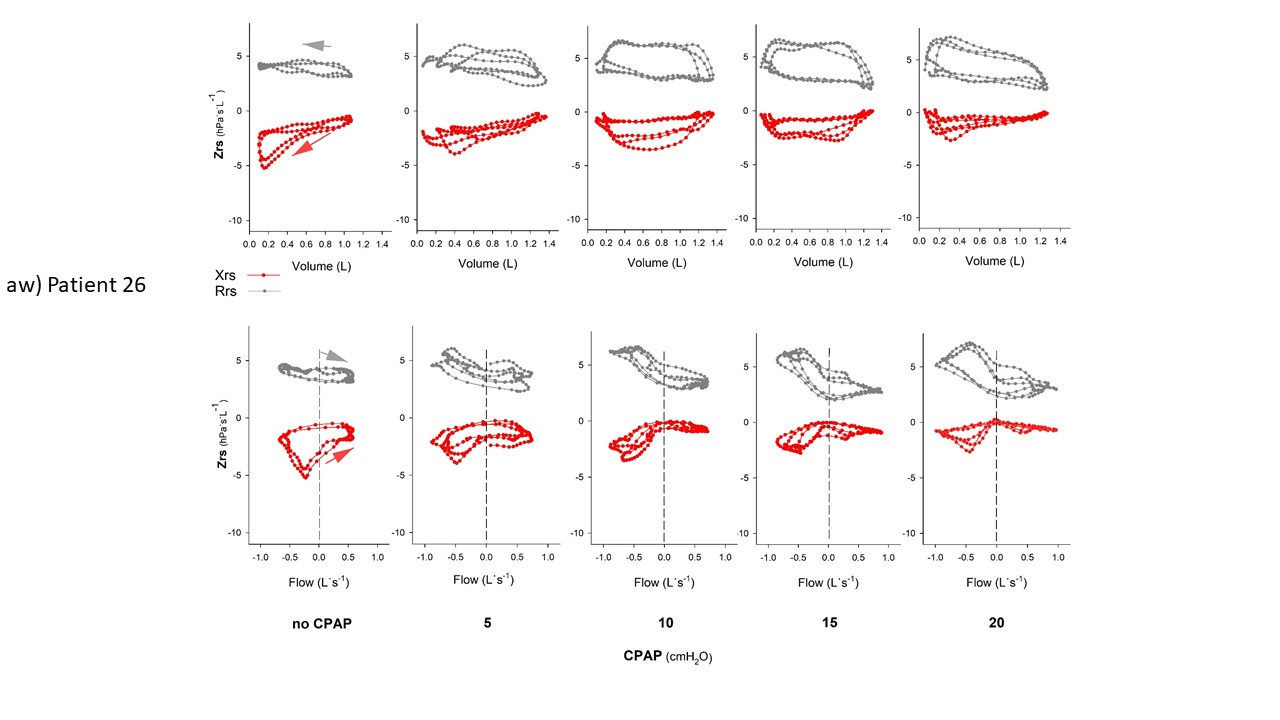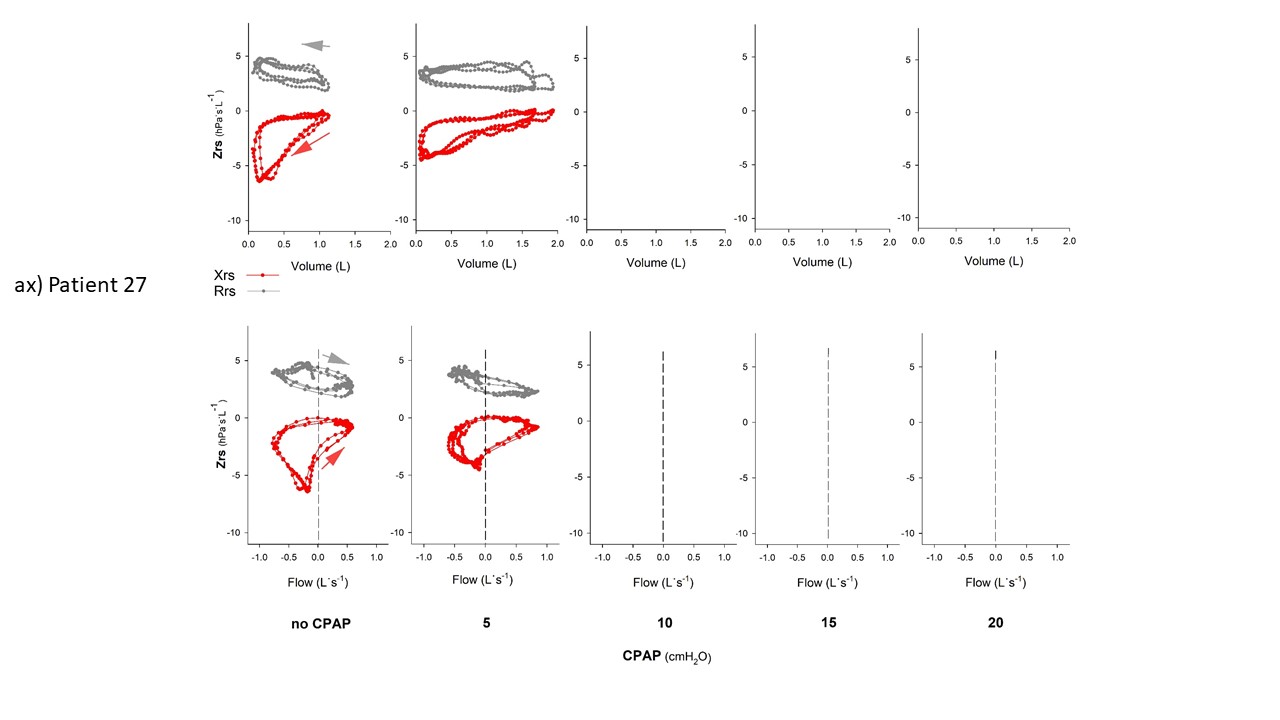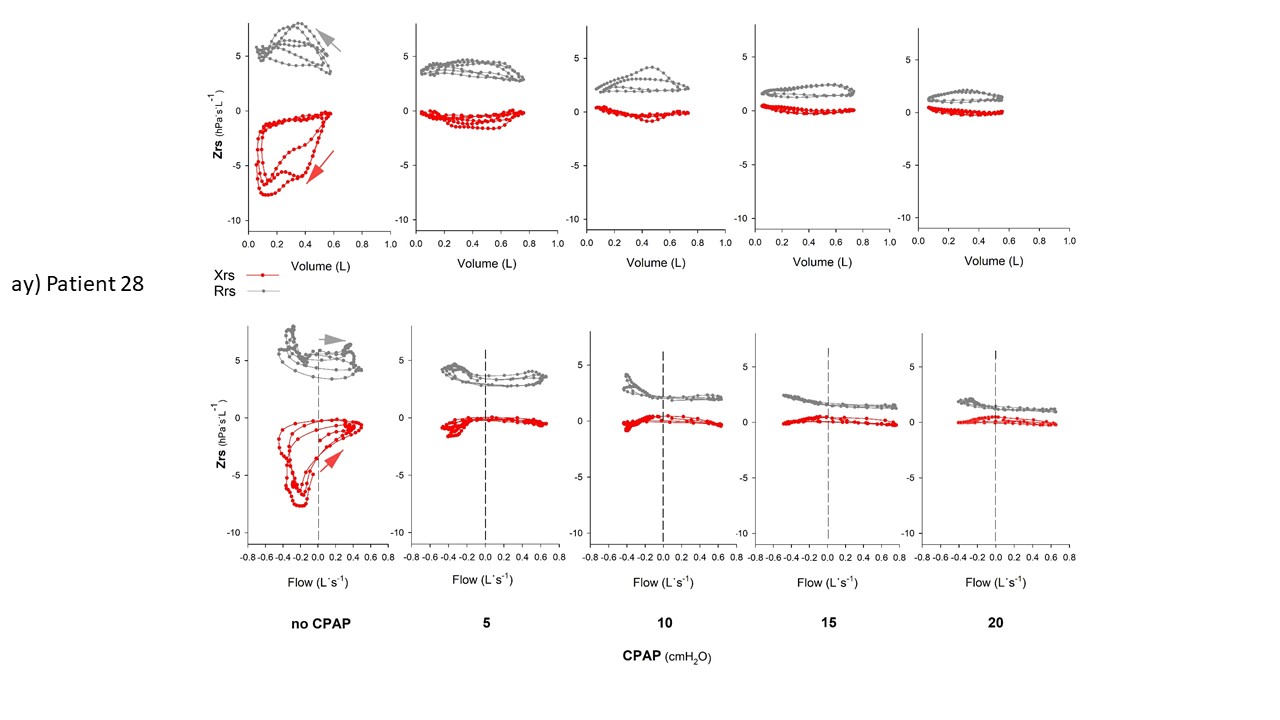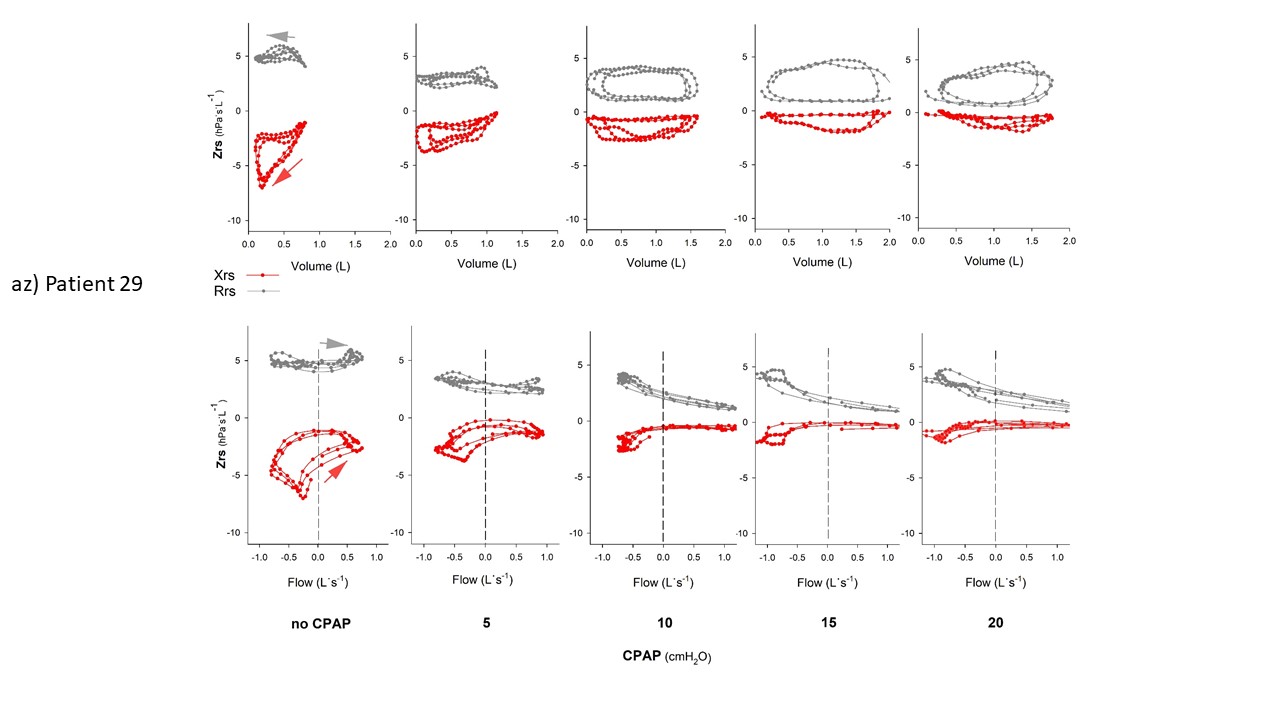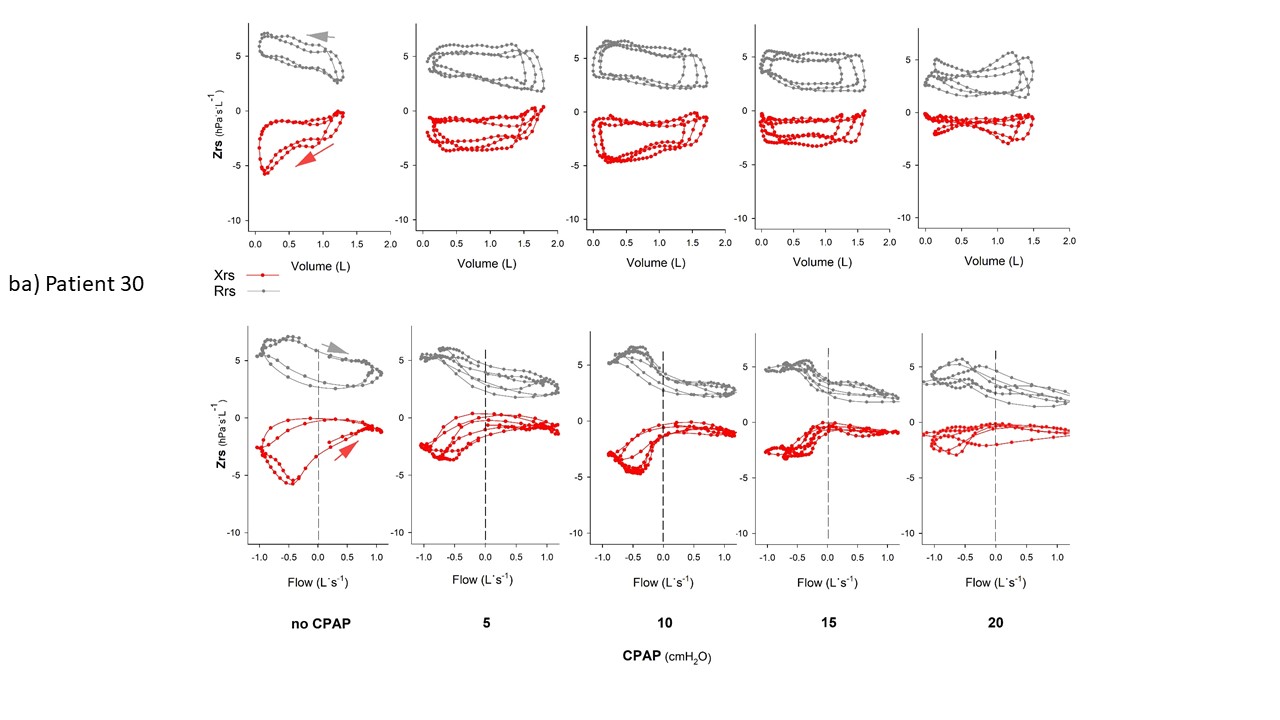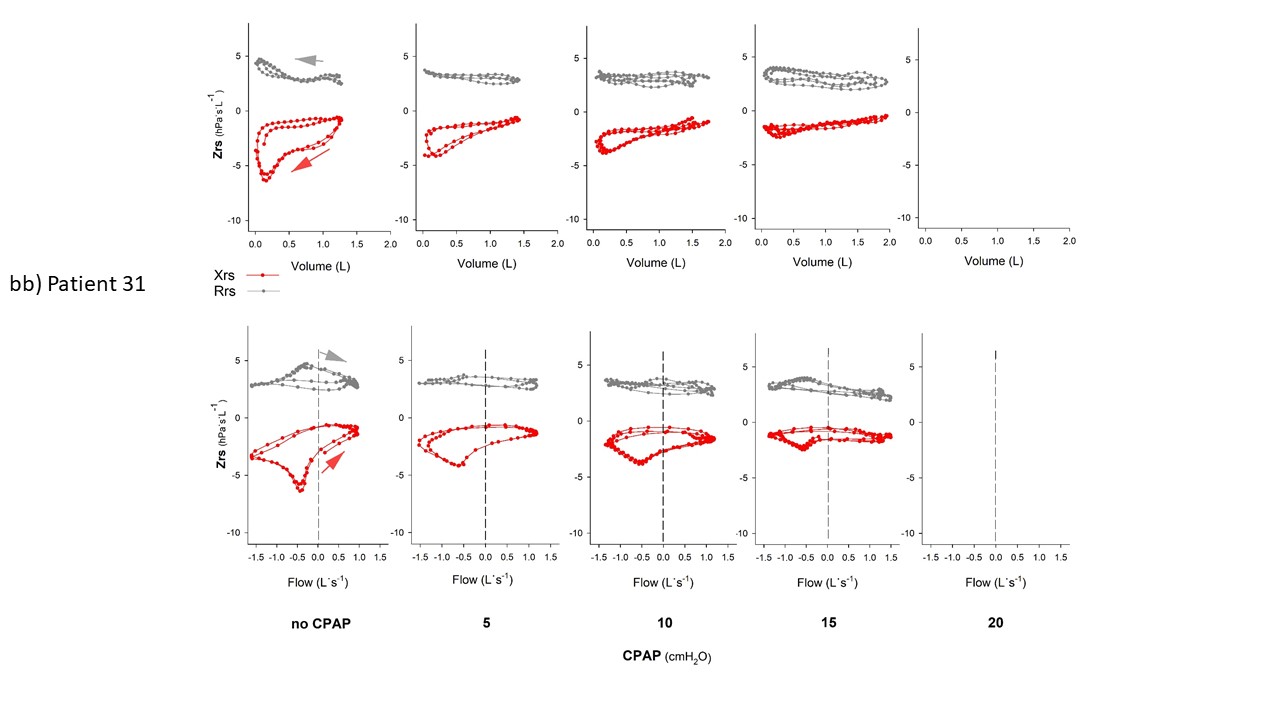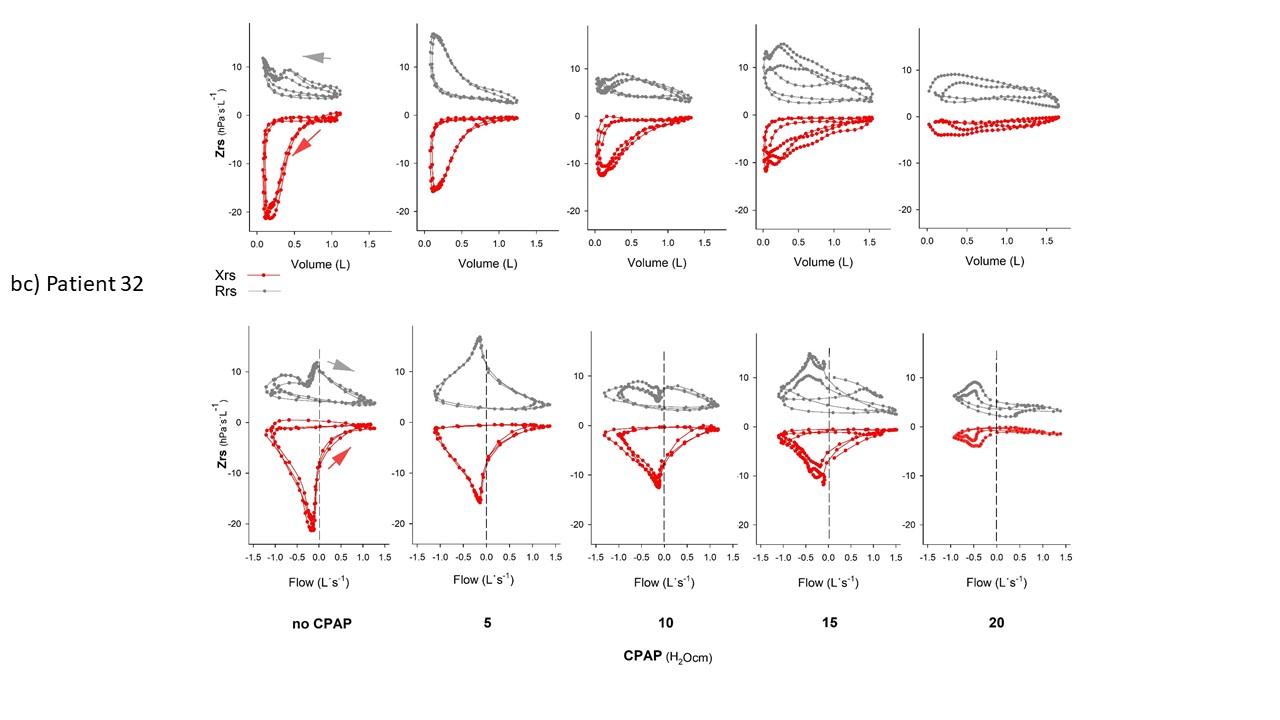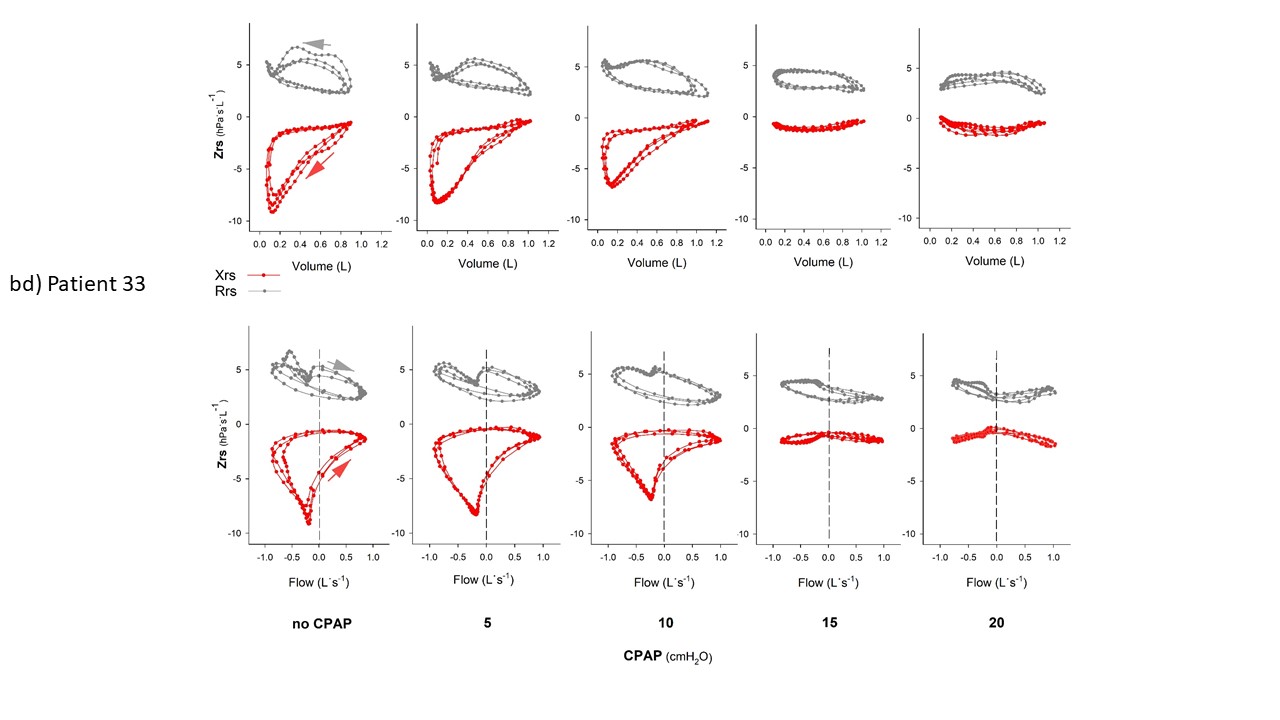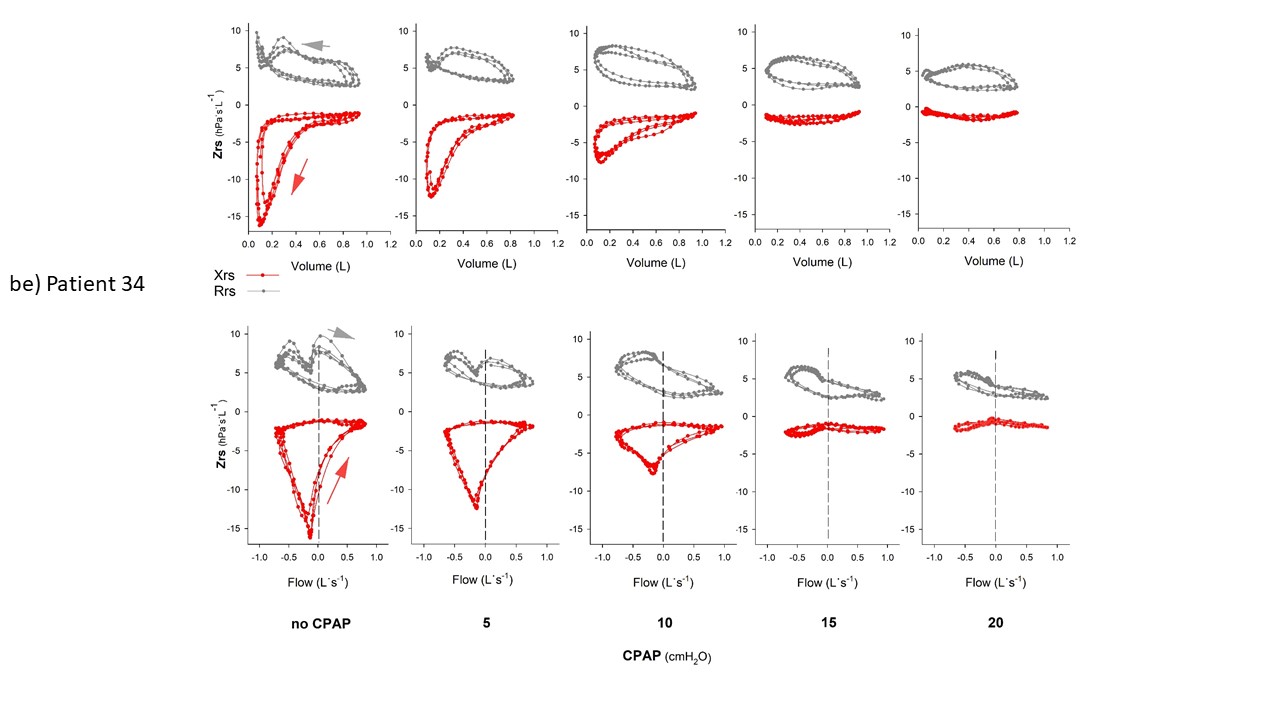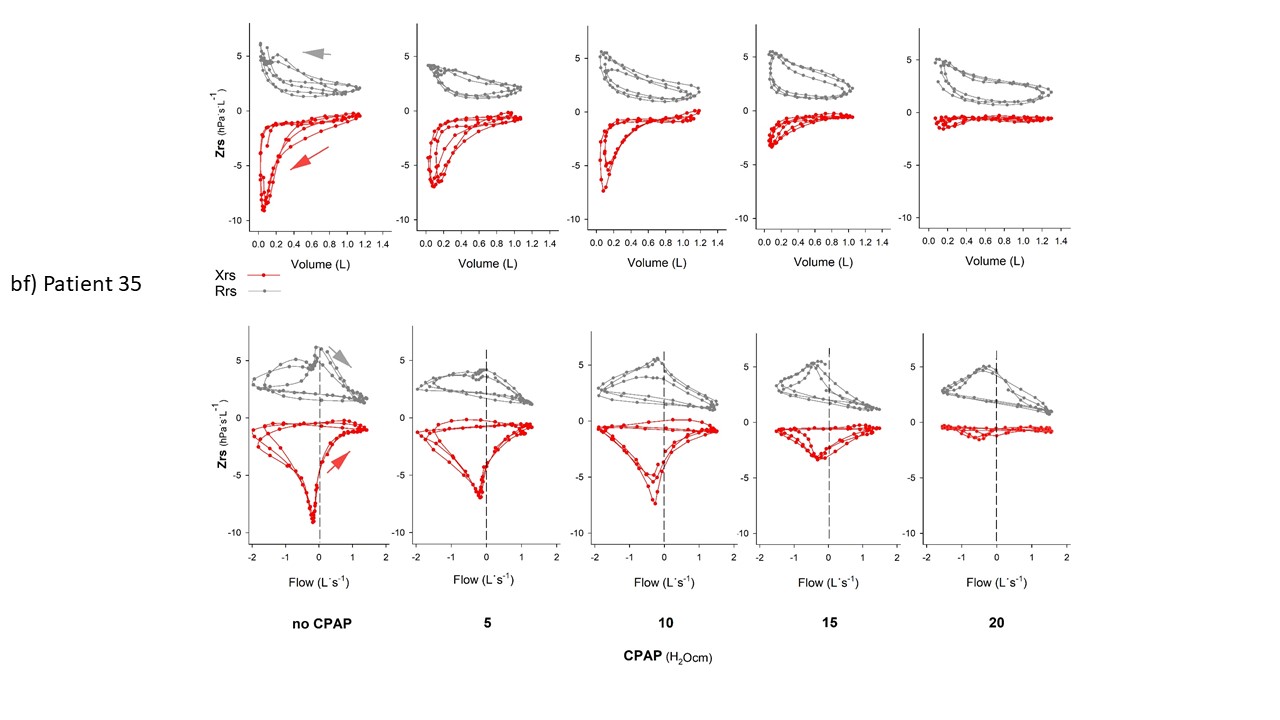 |

**References**

1. Jounieaux V, Aubert G, Dury M, et al. Effects of nasal positive-pressure hyperventilation on the glottis in normal awake subjects. Journal of applied physiology. 1995;79(1):176-85.

2. Bikov A, Pride N, Goldman M, et al. Glottal Aperture and Buccal Airflow Leaks Critically Affect Forced Oscillometry Measurements. Chest. 2015;148(3):731-8.
